# N4‐acetylcytidine in LncRNA *Gm26917* Promotes Translation in Female Germline Stem Cells by Recruiting Ribosomal Protein mRNA via EEF1A1

**DOI:** 10.1002/advs.202520059

**Published:** 2026-03-17

**Authors:** Xinyue Li, Xiaopeng Hu, Ji Wu

**Affiliations:** ^1^ Key Laboratory for the Genetics of Developmental and Neuropsychiatric Disorders (Ministry of Education) Bio‑X Institutes Shanghai Jiao Tong University Shanghai China; ^2^ Key Laboratory of Fertility Preservation and Maintenance of Ministry of Education School of Basic Medical Sciences Ningxia Medical University Yinchuan China; ^3^ Shanghai Key Laboratory of Reproductive Medicine Shanghai China

**Keywords:** long noncoding RNAs, N4‐acetylcytidine, RNA in situ conformation, sequencing, translation efficiency

## Abstract

N4‐acetylcytidine (ac4C) modification, present in both long noncoding RNAs (lncRNAs) and messenger RNAs (mRNAs), regulates cell fate decisions, yet its role in female germline stem cell (FGSC) development remains poorly understood. Here, we demonstrate that ac4C modification depletion (in vitro or in vivo) impairs FGSC maintenance by disrupting the EEF1A1‐mediated *Gm26917*‐*Rpl10* interaction, leading to attenuated protein synthesis. Reduced ac4C levels suppress FGSC viability and proliferation, dysregulate cell cycle progression, and promote differentiation and apoptosis. Integrated acRIP‐seq and RNA in situ conformation sequencing (RIC‐seq) analyses demonstrate that diminished ac4C modification in lncRNA *Gm26917* compromises its stability and weakens its interaction with *Rpl10*. Strikingly, the reduction of RPL10 is primarily caused by impaired *Gm26917*‐*Rpl10* interaction rather than direct ac4C modification. *Gm26917* or *Rpl10* knockdown disrupts FGSC fate, while ribosome profiling sequencing (Ribo‐seq) reveals that *Gm26917* depletion significantly reduces mRNA translation efficiency (TE), a defect rescued by *Rpl10* overexpression. Furthermore, EEF1A1 disruption diminishes the *Gm26917*‐*Rpl10* interaction and decreases mRNA TE. This work establishes that ac4C modification on lncRNAs governs their spatial interactions with mRNAs, elucidates a novel ac4C‐*Gm26917*‐EEF1A1‐*Rpl10* axis in FGSC maintenance, and provides a potential molecular target for modulating germ cell development and fertility.

## Graphical abstract

This work establishes that ac4C modification on lncRNA *Gm26917* governs its spatial interactions with *Rpl10* mRNA, and RBP EEF1A1 mediates the interaction between *Gm26917* and *Rpl10*. It elucidates a novel ac4C‐*Gm26917*‐EEF1A1‐*Rpl10* axis in FGSC maintenance both in vitro and in vivo, and provides a potential molecular target for modulating germ cell development and fertility.

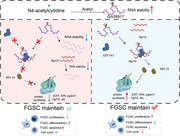

## 1 Introduction

The recent discovery of N4‐acetylcytidine (ac4C) modification in long noncoding RNAs (lncRNAs) and messenger RNAs (mRNAs) has unveiled a crucial layer of post‐transcriptional regulation in mammals [1, 2, 3]. As the sole known ac4C writer, N‐acetyltransferase 10 (NAT10) catalyzes cytidine acetylation in mRNA and lncRNA [1, 4], modulating RNA stability and translation efficiency to influence fundamental biological processes including cellular proliferation, differentiation, reprogramming, and embryonic development [5, 6, 7]. Emerging evidence highlights its functional diversity: ac4C modification enhances *Myc* mRNA translation during T cell expansion [8] and displays dynamic spatiotemporal regulation in spermatogenesis [9]. While ac4C modifications have been identified in lncRNA, their biological function significance remains poorly understood. Recent breakthroughs demonstrate that ac4C modification of lncRNA CTC‐490G23.2 promotes PTBP1‐mediated oncogenic splicing in esophageal squamous cell carcinoma (ESCC) [4], while ac4C‐stabilized lncRNA SIMALR enhances protein synthesis in macrophages [10]. These findings underscore the need for systematic investigation of ac4C‐mediated lncRNA regulation.

LncRNAs orchestrate transcriptional and post‐transcriptional control through nucleic acid and protein interactions, despite their limited protein‐coding capacity [11, 12]. Their regulatory influence extends to translation‐the pivotal stage of protein biosynthesis‐through diverse mechanisms: mRNA‐like lncRNAs may competitively inhibit ribosomal access [13], lncRNA DNAJC3‐AS1 modulates rRNA biogenesis by facilitating FBL condensation [14], and lncRNA FAM99B attenuates ribosome production via coordinately inhibiting rRNA processing and RPS29/RPL38 transcription [15]. Notably, rRNA ac4C modification governs protein synthesis via structural and functional ribosome modulation [16]. The potential crosstalk between lncRNA‐RNA interaction networks and ac4C epitranscriptomics in orchestrating ribosome biogenesis and function presents an exciting frontier for exploration.

RNA in situ conformation sequencing (RIC‐seq) represents a transformative approach for mapping spatially adjacent RNA‐RNA interaction networks mediated by RNA‐binding proteins (RBPs) [17]. The RIC‐seq offers a powerful technology to dissect how ac4C modifications shape lncRNA‐protein interactions and gene regulatory networks [10], revealing new insights into ac4C/lncRNA‐mediated post‐transcriptional control in stem cell biology.

The identification of female germline stem cells (FGSCs) has revolutionized our understanding of mammalian oogenesis, challenging the dogma of fixed ovarian reserve and offering new therapeutic potential for fertility preservation [18, 19]. FGSCs maintain a delicate balance between self‐renewal and differentiation, though the underlying regulatory mechanisms remain incompletely defined [18, 20, 21, 22]. FGSC unipotency is governed by distinct epigenetic signatures, as revealed by genome‐wide profiling of histone modifications, DNA methylation, and transcriptomes [23], and FGSCs display an adult stem cell chromatin architecture through multi‐omics integration [24]. Regulatory networks involving DNA hypomethylation [25], m6A modification [26], H3K27ac modification [27], and dynamic chromatin remodeling during meiotic initiation [28] have been implicated in FGSC maintenance. Intriguingly, Pol I transcription differentially maintains chromatin structures in human versus mouse FGSCs [29]. Despite these advances, the potential role of ac4C‐mediated epitranscriptomic regulation in FGSC development remains entirely unexplored.

This study establishes the critical role of ac4C modification in FGSC development through regulation of lncRNA *Gm26917*‐*Rpl10 m*RNA interactions. We demonstrate that ac4C levels decline markedly during FGSC differentiation, and *Nat10* ablation impaired FGSC proliferation and ovarian function. RIC‐seq analysis reveals that *N* *at* *10* knockdown disrupts EEF1A1‐mediated interaction between lncRNA *Gm26917* and *Rpl10* mRNA. Further studies show that depletion of *Nat10, Gm26917*, or *Rpl10* compromises translation efficiency (TE), leading to proliferation arrest, cell cycle defects, and enhanced differentiation/apoptosis in FGSCs. These findings elucidate a novel ac4C‐mediated regulatory mechanism in FGSCs development and provide fundamental insights into epitranscriptomic control of germ cell biology.

## 2 Results

### 2.1 Dynamic Reduction of ac4C Modification During FGSC Differentiation

**FIGURE 1:**
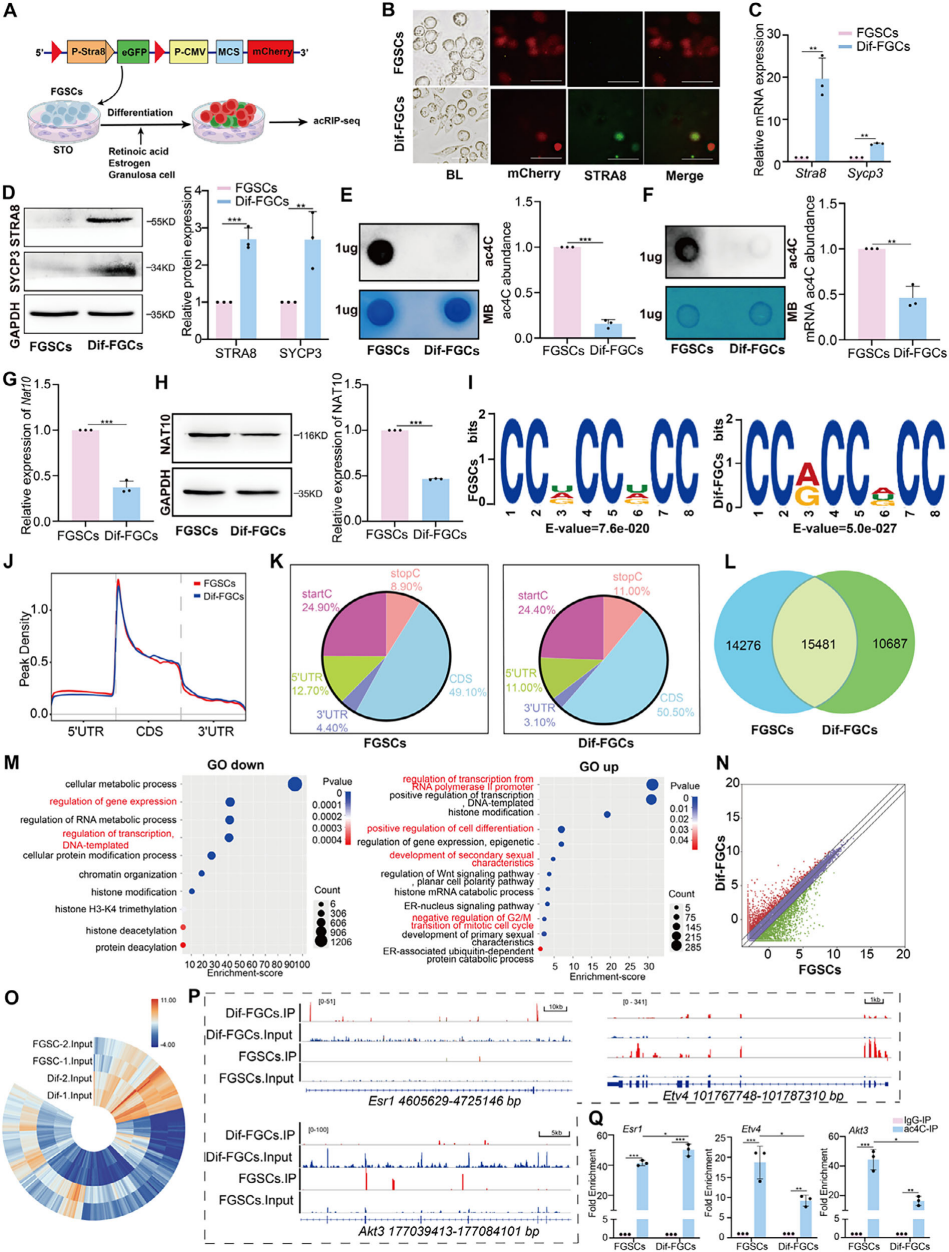
Dynamics of ac4C modifications in FGSCs and Dif‐FGCs. (A) Schematic illustration of the dual‐fluorescence lentiviral (DFL) reporter system (mCherry‐STRA8‐promoter‐eGFP) for monitoring FGSC differentiation. (B) Bright‐field and fluorescence microscopy images depicting morphological changes in FGSCs and Dif‐FGCs. (C and D) Validation of FGSC differentiation by RT‐qPCR (C) and western blotting (D) analysis of meiotic markers (STRA8 and SYCP3). (E and F) Dot blotting quantification of ac4C modifications in total RNA (E) and poly(A) + mRNA (F) from FGSCs and Dif‐FGCs, normalized by methylene blue (MB) staining. ac4C was detected by antibody staining followed by HRP‐based chemiluminescence. (G and H) NAT10 expression levels assessed by RT‐qPCR (G) and western blotting (H) with quantitative analysis. (I) Consensus sequence motifs enriched at ac4C modification sites identified by acRIP‐seq. (J) Density distribution of ac4C peaks along mRNA transcripts (5’→3’). (K) Genome‐wide localization of ac4C peaks across mRNA features (CDS, 3’/5’‐UTRs, start/stop codons). (L) Differential ac4C peaks between FGSCs and Dif‐FGCs. (M) GO enrichment of genes with differential ac4C modification genes (downregulated: left; upregulated: right). (N) Volcano plot of transcriptomic changes (red: upregulated; green: downregulated; gray: nonsignificant). (O) Heatmap integrating DEGs and differential ac4C‐modified transcripts. (P and Q) IGV browser tracks (P) and acRIP‐qPCR validation (Q) of ac4C modifications in *Esr1*, *Etv4*, and *Akt3*. Data represent mean ± SD (acRIP‐seq: n = 2 biological replicates; other results: n = 3 biological replicates). ^*^ *p* < 0.05, ^**^ *p* < 0.01, ^***^ *p* < 0.001. Fold change>1.5 and p‐value ≤ 0.05.

The FGSCs used in this study were rigorously validated (Figure S1A, B). To enable real‐time monitoring of differentiation, we established an mCherry‐STRA8‐promoter‐GFP dual‐reporter system (Figure 1A), where mCherry served as a constitutive marker while GFP expression was driven by the meiosis‐specific STRA8 promoter upon differentiation (Figure 1B). Following our established protocol, differentiated female germ cells (Dif‐FGCs) exhibited robust induction of meiotic markers STRA8 and SYCP3 at both mRNA and protein levels, which were undetectable in undifferentiated FGSCs (Figure 1C, D). This confirmed the efficacy of our differentiation system for subsequent studies.

To investigate ac4C's role in FGSC differentiation, we quantified its modification levels. RNA dot blotting revealed a significant reduction of ac4C modifications in total RNA (Figure 1E) and poly(A)+ mRNA (Figure 1F) from Dif‐FGCs compared to FGSCs. Consistently, both mRNA and protein expression of NAT10, the sole known ac4C “writer”, were markedly downregulated in Dif‐FGCs (Figure 1G, H), aligning with the observed ac4C modification decrease. Acetylated RNA immunoprecipitation and sequencing (acRIP‐seq) profiling identified the canonical CXXCXXCX ac4C motif in both FGSCs and Dif‐FGCs (Figure 1I). mRNA exited widespread distribution of ac4C modification, with the highest density observed in the coding sequences (CDS) region (Figure 1J). Comparative analysis revealing dynamic redistribution during differentiation, CDS regions exhibited a 1.4% increase in ac4C peak density (from 49.1% to 50.5%), stop codon‐proximal regions showed a 2.1% increase (from 8.9% to 11%), the start codon remained relatively stable (24.9% vs. 24.4%), while reductions of 1.3% (4.4% to 3.1%) and 1.7% (12.7% to 11%) were observed in the 3'‐UTR and 5'‐UTR regions, respectively (Figure 1K). Notably, while 15,481 ac4C peaks were shared between FGSCs and Dif‐FGCs, 14,276 and 10,687 peaks were unique to each state, respectively (Figure 1L).

Gene Ontology (GO) analysis of differentially modified genes highlighted enrichment in gene expression regulation, positive regulation of cell differentiation, and negative regulation of G2/M cell cycle transition (Figure 1M). Kyoto Encyclopedia of Genes and Genomes (KEGG) pathway analysis further associated these genes with mTOR, AMPK, Hippo signaling, and pathways regulating pluripotency (Figure S1C). Transcriptomics analysis (Input data) identified differentially expressed genes (DEGs) (Figure 1N), and combined analysis with ac4C modification data yielded 124 upregulated and 201 downregulated DEGs (Figure 1O). Intersection with ac4C peaks revealed differentiation‐linked changes, ac4C modification increased at *Esr1* and decreased at *Etv4* and *Akt3* loci (Figure 1P), a trend validated by acRIP‐qPCR (Figure 1Q) and consistent with the corresponding expression change (Figure S1D). Furthermore, treatment with Remodelin (RE), a NAT10‐specific inhibitor, significantly impaired FGSC viability and proliferation (Figure S2A, B). Strikingly, RE induced FGSC differentiation, as evidenced by elevated STRA8 and SYCP3 expression (Figure S2C, D), underscoring the role of ac4C modification in maintaining FGSC undifferentiated state.

### 2.2 ac4C Modification Governs FGSC Proliferation, Cell Cycle, Differentiation, and Apoptosis in Vitro

**FIGURE 2:**
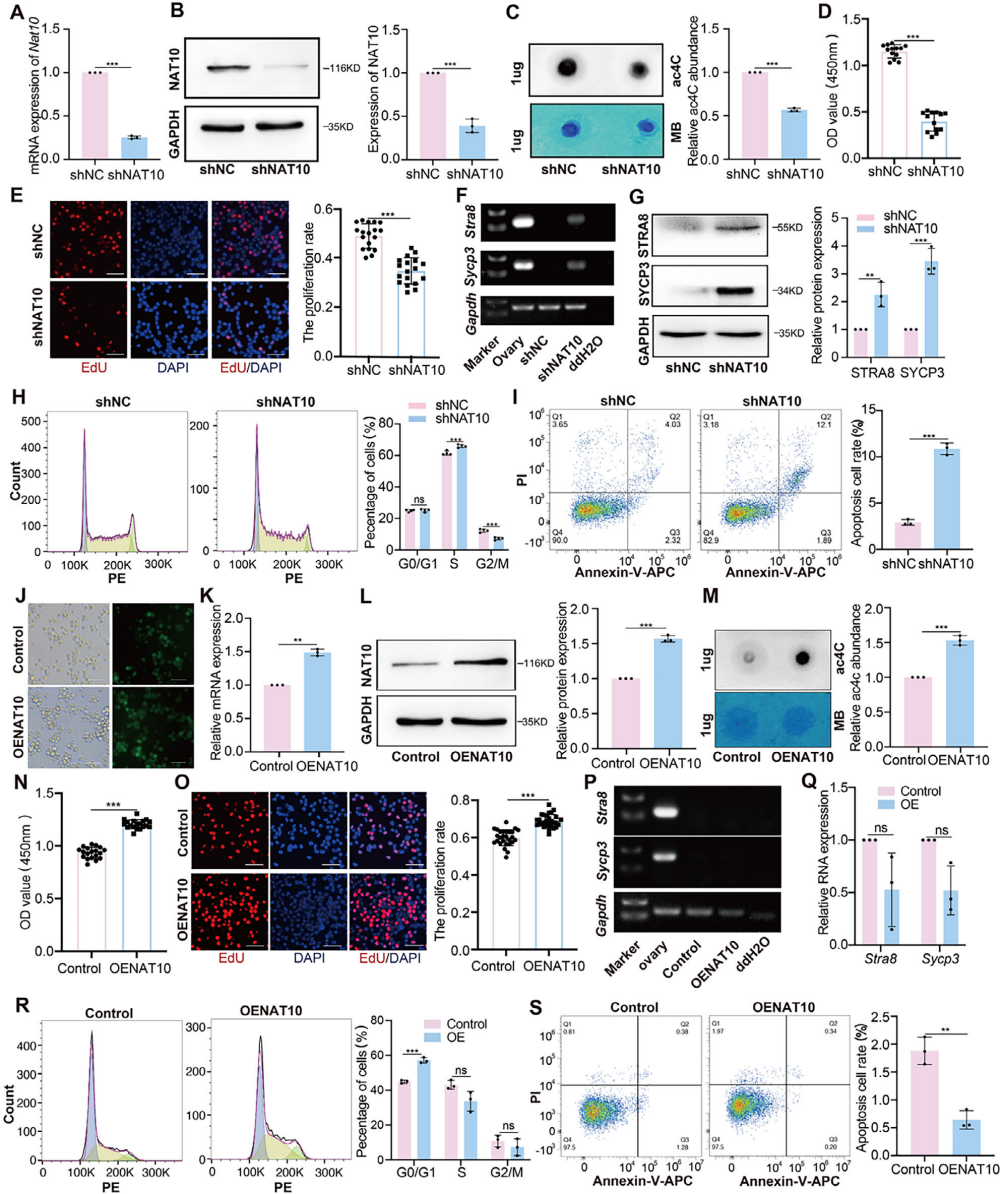
ac4C modification regulates FGSC differentiation, cell cycle, and apoptosis. (A and B) Validation of *N* *at* *10* knockdown efficiency by RT‐qPCR (A) and western blotting (B). (C) Dot blotting analysis of global ac4C modifications in shNC and shNAT10 FGSCs. (D and E) CCK8 detected cell viability (D) and EdU detected cell proliferation (E), both of these were significantly decreased after shNAT10. (F and G) Downregulation of meiotic markers (STRA8, SYCP3) post‐*N* *at* *10* knockdown via RT‐qPCR (F) and western blotting (G). (H) Cell cycle phase distribution analyzed by flow cytometry (shNC vs. shNAT10). PE: PI fluorescence detected in the PE channel. (I) Increased apoptosis rate in shNAT10 FGSCs (annexin V/PI staining). (J) FGSCs were infected with *Nat10* overexpression control and *Nat10* overexpression lentivirus. (K and L) Verification of *N* *at* *10* overexpression by RT‐qPCR (K) and western blotting (L). (M) Enhanced ac4C modifications in OENAT10 FGSCs (Dot blotting). (N and O) Functional effects of *N* *at* *10* overexpression, including increased cell viability (CCK‐8, N) and enhanced proliferation (EdU, O). (P and Q) Detection the expression of *Stra8* and *Sycp3* in OENAT10 FGSCs by RT‐PCR (P) and RT‐qPCR (Q). (R) Detection of cell cycle in OENAT10 FGSCs by flow cytometry. (S) Reduced apoptosis rate in OENAT10 FGSCs (flow cytometry). Scale bars: 50 µm. ^**^ *p* < 0.01, ^***^ *p* < 0.001. Data represent mean ± SD (n = 3 biological replicates).

To elucidate the functional consequences of ac4C modification in FGSC biology in vitro, we employed lentiviral‐mediated *Nat10* knockdown (shNAT10) to reduce ac4c modification (Figure S3A, B). Quantitative analysis confirmed efficient NAT10 depletion at both transcript and protein levels (Figure 2A, B). The global ac4C modification levels were significantly decreased in shNAT10 compared with shNC FGSCs (Figure 2C), along with a significant reduction in cell viability and cell proliferation (Figure 2D, E).

Strikingly, NAT10 depletion triggered FGSC differentiation, as evidenced by upregulation of STRA8 and SYCP3 at both transcriptional and translational levels (Figure 2F, G). Cell cycle profiling revealed a profound S‐phase arrest concomitant with reduced G2/M‐phase following shNAT10, consistent with depressed cell proliferation (Figure 2H). Cell cycle‐related protein expressions of cyclin B1, cyclin D1, and CDK4 were significantly up‐regulated, while the expression of cyclin A2 was significantly down‐regulated, after shNAT10 (Figure S3C). Notably, shNAT10 induced apoptosis (Figure 2I), supported by elevated cleaved PARP/ cleaved caspase‐3/BAX and diminished BCL‐2 expression (Figure S3D).

In contrast, *Nat10* overexpression (OENAT10) significantly increased RNA ac4C modification level (Figure 2J–M), and cell viability and cell proliferation were significantly increased (Figure 2N, O). The expression of *Stra8* and *Sycp3* are no difference between Control and OENAT10 (Figure 2P, Q). Cell cycle demonstrated a pronounced accumulation of G0/G1‐phase populations (Figure 2R), and the apoptotic rate was decreased after OENAT10 (Figure 2S). Given NAT10's high basal expression level, and considering that shNAT10 elicited robust phenotypic changes (differentiation and apoptosis), we employed knockdown models for mechanistic investigations. This loss‐of‐function strategy avoided saturation artifacts while ensuring detectable phenotypic outputs.

### 2.3 ac4C Acetyltransferase Deficiency Impairs FGSC Proliferation and Causes Mouse Infertility

**FIGURE 3:**
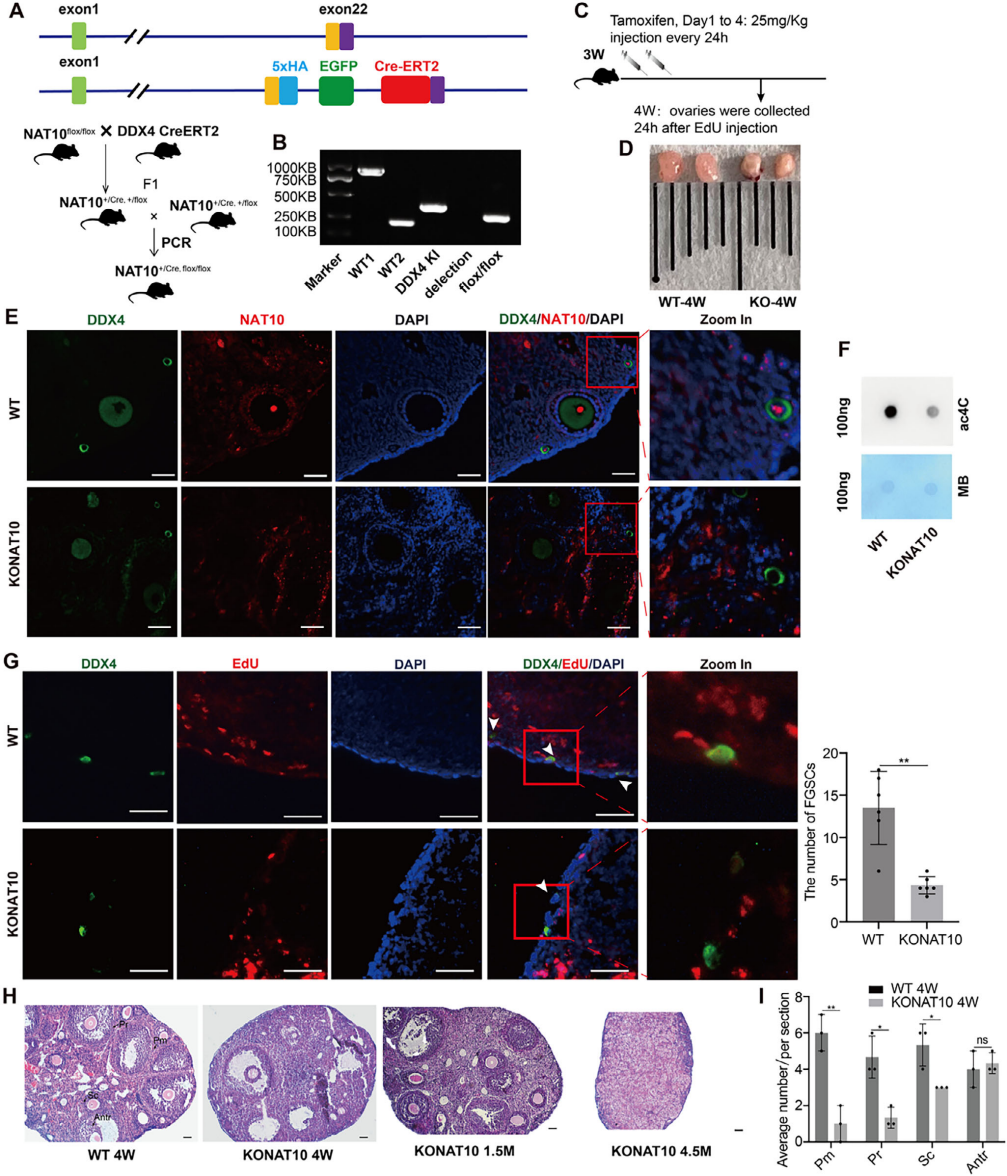
NAT10 knockout significantly reduces FGSC population and impairs ovarian development. (A) Schematic of the conditional knockout (KO) mouse model design. (B) Genotype verification by RT‐PCR. (C) Experimental timeline for mouse model analysis. (D) Gross morphological comparison of ovaries from WT and KONAT10 mice. (E) Validation of NAT10 knockout efficiency by dual immunofluorescence staining for DDX4 (green) and NAT10 (red), and nuclear counterstain with DAPI (blue). (F)  Dot blotting analysis of ac4C modifications in WT and KONAT10 mice germ cells. (G) Quantification of FGSC population by co‐staining of germ cells (DDX4, green) and proliferating cells (EdU, red), and DAPI nuclear counterstain (blue). White arrows indicate double‐positive cells, FGSCs. (H) Histological analysis of ovarian development: H&E staining of ovarian sections from KONAT10 mice at 4 W, 1.5 M, and 4.5 M. (I) Enumeration and classification of follicles in 4 W ovaries: primordial follicles (Pm), primary follicles (Pr), secondary follicles (Sc), antral follicles (Antr). Scale bars: 50 µm. ^*^ *p* < 0.05, ^**^ *p* < 0.01; ns: not significant. Data represent mean ± SD (n = 6 biological replicates).

To elucidate the role of ac4C modification in FGSC development in vivo, we generated a conditional *Nat10* knockout mouse model (DDX4creERT2; NAT10flox/flox). In this system, tamoxifen‐inducible Cre recombinase expression under the DDX4 promoter enabled germ cell‐specific NAT10 ablation (Figure 3A). Genotyping by RT‐PCR confirmed the successful generation of DDX4+/cre, NAT10flox/flox mice (Figure 3B), and the experimental timeline is outlined in Figure 3C. Phenotypically, KONAT10 ovaries were markedly smaller, exhibited a pale, dull appearance compared to WT ovaries (Figure 3D). Immunofluorescence of ovarian paraffin sections revealed robust NAT10 expression colocalized with DDX4‐positive germ cells in WT mice, whereas KONAT10 germ cells showed complete loss of NAT10 signal, validating efficient germline‐specific deletion (Figure 3E), and we found a significant reduction in RNA ac4C modification within KONAT10 germ cells (Figure 3F).

To evaluate KONAT10 effects on FGSCs dynamics, we performed dual DDX4/EdU labeling on ovarian frozen sections, leveraging EdU incorporation to identify proliferating FGSCs, based on their germ stem cell characteristics [30]. FGSCs (DDX4+EdU+) were predominantly localized to near the ovarian cortical surface. Strikingly, KONAT10 ovaries exhibited a significant reduction in FGSC numbers, indicating impaired proliferation upon *Nat10* depletion (Figure 3G). Consistent with this, KONAT10 female mice failed to produce offspring after prolonged (3 m) mating with WT males. Histological analysis (H&E staining) of KONAT10 ovaries at 4 weeks (4 W), 1.5 months (1.5 m), and 5.5 months (5.5 M) post‐induction revealed progressive follicular depletion, underscoring severe ovarian dysfunction (Figure 3H). Quantitative assessment demonstrated significant declines in primordial, primary, and secondary follicle counts in 4 W KONAT10 ovaries versus WT, while antral follicles remained unaffected (Figure 3I). These data are consistent with the observed FGSC proliferation defect. Taken together, NAT10 ablation in FGSCs disrupts their proliferative capacity, leading to aberrant folliculogenesis, ovarian failure, and ultimately infertility.

### 2.4 RIC‐seq Reveals Diminished lncRNA *Gm26917*‐*Rpl10* mRNA Interaction Upon Inhibition of ac4C Modification

Based on preliminary experimental data, the observed ac4C dynamics on differentiation‐associated genes (Figure 1Q) cannot fully explain the pleiotropic phenotypes in FGSC development (Figure 2D–I). To determine whether ac4C modification regulates FGSC development through RNA‐RNA interactions, we performed RIC‐seq in shNC and shNAT10 FGSCs. Replicate samples showed high reproducibility (Figure S4A), and paired reads were filtered for downstream analysis (Figure S4B). The ratio of inter‐, intramolecular chimeric reads in the intron and exon region, the percentage of intron is relatively higher than exon, suggesting a predominant role for intron region RNA‐other RNA interaction (Figure S4C). High‐confidence interactions were identified by comparing observed pairs to simulated random counts (Figure S4D). Strong conservation was observed between shNC and shNAT10 binding motifs (Figure S4E). Parallel Long RNA‐seq of shNC and shNAT10 FGSCs showed robust intra‐group correlation (Figure S4F). Gene expression was normalized using Fragments Per Kilobase Million (FPKM) (Figure S4G), and integrated analysis of DEGs (Figure S4H) and RNA interaction networks identified hub RNAs (Figure S4I), with further characterization of their genomic distribution (Figure S4J).

**FIGURE 4:**
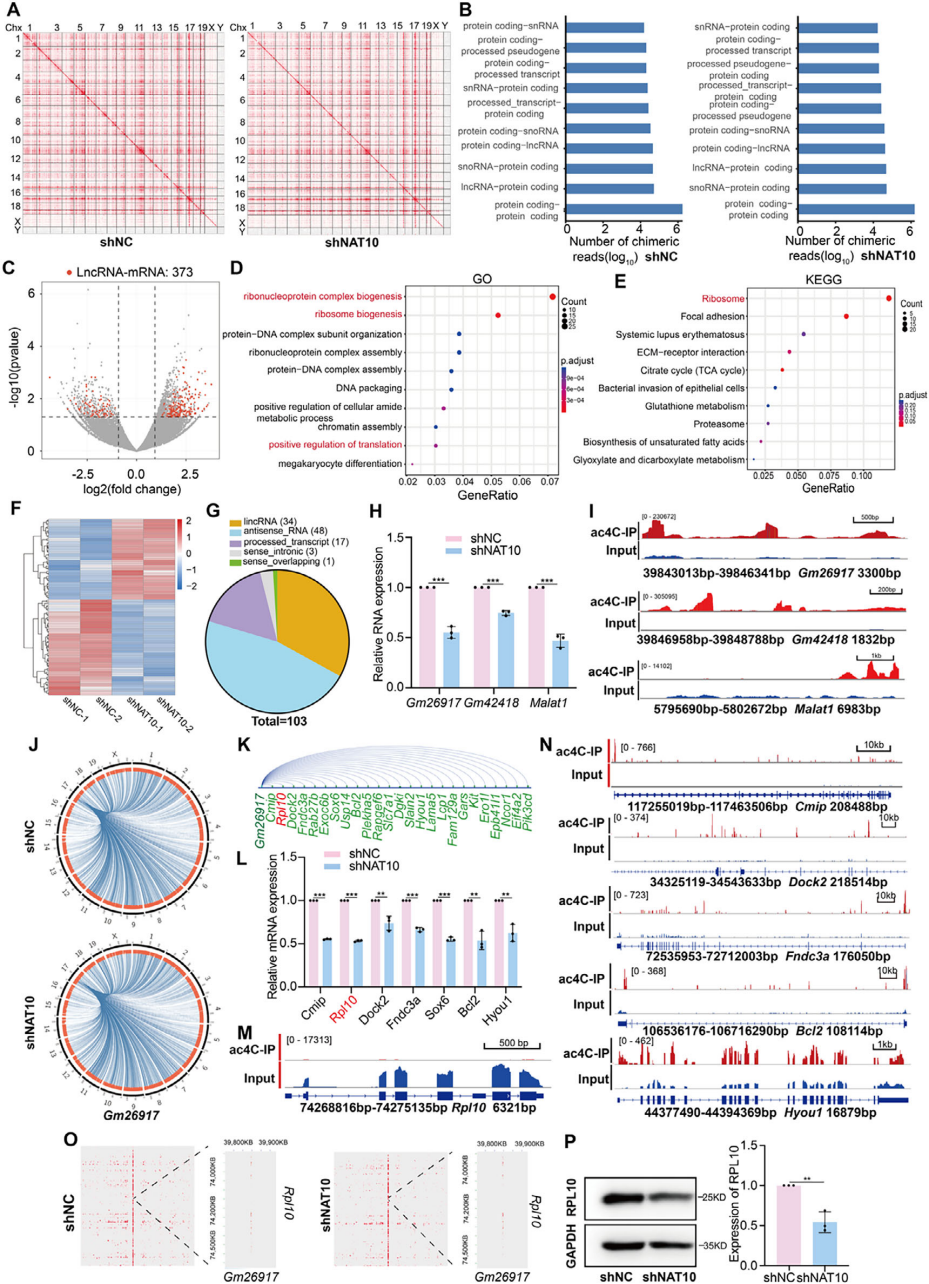
Integrated analysis of RIC‐seq and long RNA‐seq identifies *Gm26917*‐*Rpl10* mRNA interactions. (A) Chromosome‐wide RNA‐RNA interaction matrix heatmap. (B) Distribution of RIC‐seq chimeric reads by interaction type in shNC vs. shNAT10 groups. (C) Volcano plot of differentially interacting RNA pairs (shNC vs. shNAT10). The red dots: lncRNA‐mRNA. (D and E) GO (D) and KEGG (E) pathway enrichment of differential RNA‐RNA interactions. (F) Differentially expressed lncRNAs (shNC vs. shNAT10). Y‐axis, gene names; X‐axis, sample names; each tile represents the normalized expression value of each gene across different samples. (G) Classification of differential lncRNAs in FGSCs. (H) RT‐qPCR validation of lncRNAs (*Gm26917*, *Gm42418*, *Malat1*). (I) ac4C‐seq IGV tracks for *Gm26917*, *Gm42418*, and *Malat1* in FGSCs. (J) Circos plot of *Gm26917* RNA interactome: outer rings show chromosomes; inner rings display normalized interaction frequencies. (K) Differential mRNA partners of *Gm26917* (shNC vs. shNAT10). (L) RT‐qPCR validation of mRNAs in (K). (M) ac4C‐seq IGV tracks for *Rpl10*. (N) ac4C‐seq IGV tracks for *Cimp*, *Dock2*, *Fndc3a*, *Bcl2*, and *Hyou1*. (O) Heatmap of *Gm26917*‐*Rpl10* interaction signals. (P) Western blotting analysis of RPL10 protein levels. ^**^ *p* < 0.01, ^***^ *p* < 0.001 (Student's t‐test; mean ± SD; Long RNA‐seq and RIC‐seq: n = 2 biological replicates; other results: n = 3 biological replicates). Threshold: fold change>1.2, p ≤ 0.05.

Global RNA interaction heatmaps highlighted distinct profiles between shNC and shNAT10 FGSCs (Figure 4A). While protein‐coding self‐interactions (which were predominantly mRNA self‐interactions, RIC‐seq data) dominated, lncRNA–mRNA interactions were the second most abundant class (Figure 4B). A volcano plot revealed differentially interacting RNA pairs, with lncRNA‐mRNA interactions accounting for approximately one‐quarter (373/1268) of all differential interaction pairs (Figure 4C). GO/KEGG analysis implicated ribosome biogenesis, chromatin assembly, and translational regulation (Figure 4D, E). Focusing on lncRNA‐mRNA interactions, we identified 56 downregulated and 47 upregulated lncRNAs (Figure 4F), and statistical classification and quantification of lncRNAs (Figure 4G). qPCR validated reduced expression of lncRNAs *Gm42418*, *Gm26917*, and *Malat1* in shNAT10 FGSCs (Figure 4H). Notably, *Gm26917* exhibited significant ac4C peak enrichment (Figure 4I) and the most extensive RNA interactome (15,755 in shNC vs 16,659 in shNAT10) (Figure 4J), suggesting its broad regulatory role. Based on a comprehensive evaluation of the experimental data, *Gm26917* was selected as the candidate for subsequent studies. Screening for mRNAs with both decreased *Gm26917* binding and significant downregulation upon shNAT10 (Figure 4K, L). Unlike other partners, *Rpl10* lacked detectable ac4C peaks in IP fractions (Figure 4M, N), implicating a unique regulatory mechanism. The *Gm26917*‐*Rpl10* interaction emerged as a central network node, with their interaction heat map displayed in Figure 4O. And RPL10 expression was significantly reduced upon shNAT10 (Figure 4P).

### 2.5 The ac4C‐*Gm26917*‐*Rpl10* Axis Governs FGSC Maintenance Through Epitranscriptomic Regulation

**FIGURE 5:**
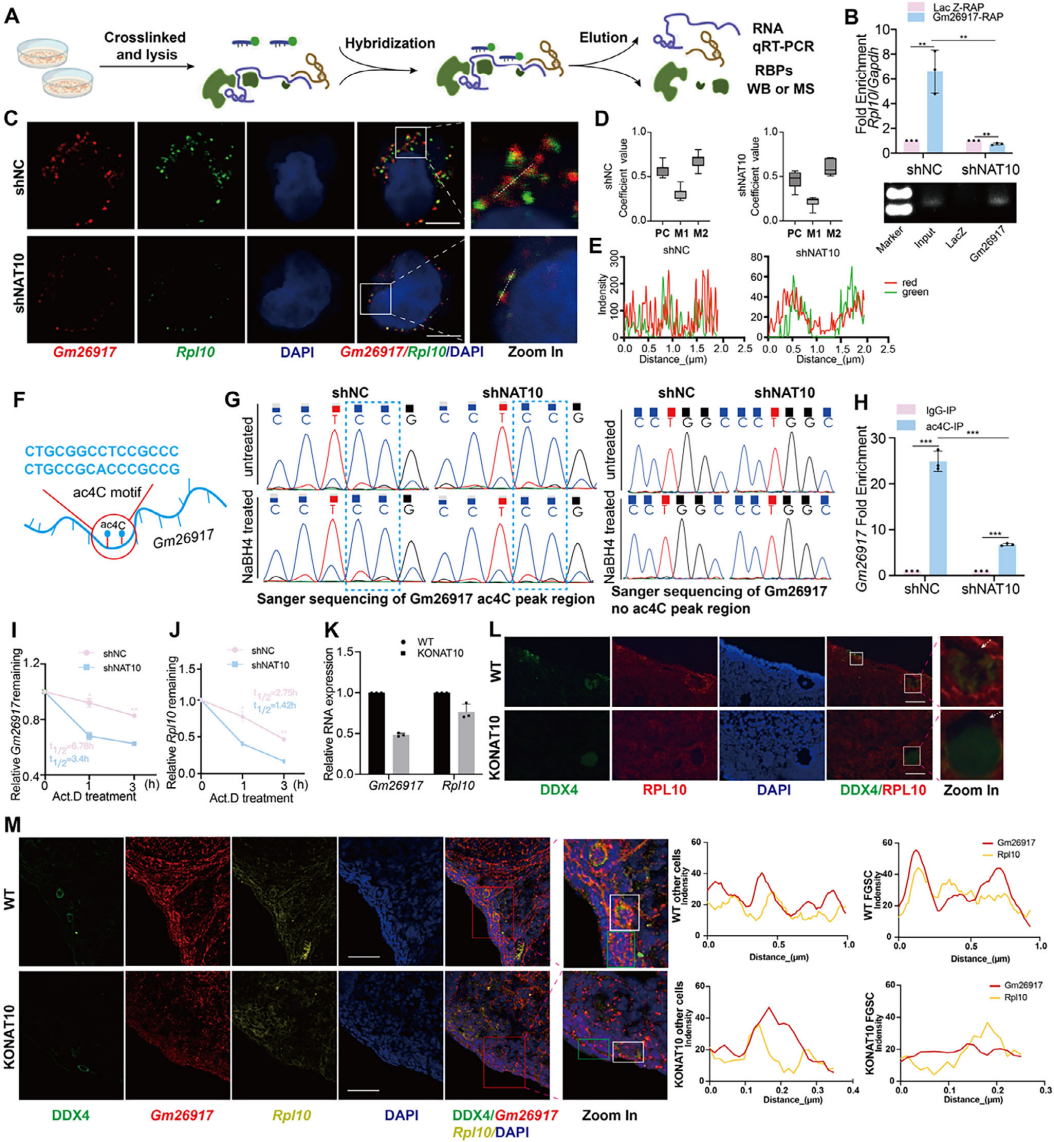
Decreased ac4C modification disrupts *Gm26917*‐*Rpl10* interaction. (A) RAP assay schematic. (B) RAP‐qPCR showing *Rpl10* binding to *Gm26917* probes (agarose gel: *Rpl10* product detected). (C) Confocal imaging of *Gm26917* (red)/*Rpl10* (green) co‐localization (DAPI: blue; insets: magnified regions). (D) Pearson's and Manders’ coefficients (M1thr and M2thr) were analyzed by JACoP. PC: Pearson's coefficients: which is close to 1 as red and green channel intensity distributions are linked; M1: M1thr, represents the fraction of *Gm26917* signals overlapping with *Rpl10*; M2: M2thr, represents the fraction of *Rpl10* signals overlapping with *Gm26917*. N = 6 cells. (E) Fluorescence intensity along the line shown in Figure C was quantified for overlapping foci (yellow). (F) Potential ac4C consensus motif in *Gm26917*. (G) Sanger sequencing of *Gm26917* ac4C peaks (green: C‐to‐T mutation) and *Gm26917* no ac4C peak region. (H) ac4C RIP‐qPCR detectes ac4C modification of *Gm26917*. (I) Reduced *Gm26917* half‐life upon shNAT10. (J) Decreased *Rpl10* mRNA stability upon shNAT10. (K) RT‐qPCR analysis of gene expression in WT and KONAT10 ovaries. (L) Co‐staining of DDX4 (red) and RPL10 (green) in ovarian sections. White boxs and rows: germ cells. (M) Co‐localization of *Gm26917* (red) and *Rpl10* (yellow) in ovarian sections. White/green boxes: germ/non‐germ cell regions for quantification. Scale bars: 10 µm (C); Scale bars: 50 µm (L and M). ^*^ *p* < 0.05, ^**^ *p* < 0.01, ^***^ *p* < 0.001; ns: not significant. Data: mean ± SD (n = 3 biological replicates).

To mechanistically dissect the role of ac4C modification in FGSC biology, we first validated the physical interaction between lncRNA *Gm26917* and *Rpl10* mRNA using RAP (RNA antisense purification)‐qPCR. Notably, shNAT10 FGSCs (with impaired ac4C deposition) exhibited significantly reduced *Gm26917*‐*Rpl10* interaction compared to shNC controls (Figure 5A, B). Furthermore, smFISH co‐localization confirmed their perinuclear cytoplasmic association (Figure 5C–E). Next, computational analysis via the PACES database (http://www.rnanut.net/paces/) identified two evolutionarily conserved ac4C motifs in *Gm26917* (positions 2684–2699 and 2711–2725; Figure 5F; and S5A), which were biochemically validated through borohydride‐induced C to T mutation during Sanger sequencing (Figure 5G and S5B). NAT10 depletion caused a pronounced reduction in *Gm26917* ac4C modification (Figure 5H), leading to transcript destabilization (6.78 to 3.4 h; Figure 5I). Intriguingly, although *Rpl10* mRNA lacks detectable ac4C sites, its half‐life was similarly compromised (2.75 to 1.42 h; Figure 5J), suggesting *Gm26917* acts as a trans‐stabilizer of *Rpl10*. Consistent with the cellular experimental findings, the expression levels of *Gm26917* and *Rpl10* were significantly reduced in ovarian germ cells of KONAT10 mice (Figure 5K). In frozen sections of mouse ovaries, germ cells were localized by the germ cell marker DDX4. Within the germ cells of KONAT10 mice, the fluorescence signal intensity of RPL10 protein was diminished (Figure 5L), and the co‐localization signal intensity of lncRNA *Gm26917* with *Rpl10* mRNA was also decreased (Figure 5M). Together, these results collectively confirm that this regulatory axis is also operative in vivo.

**FIGURE 6:**
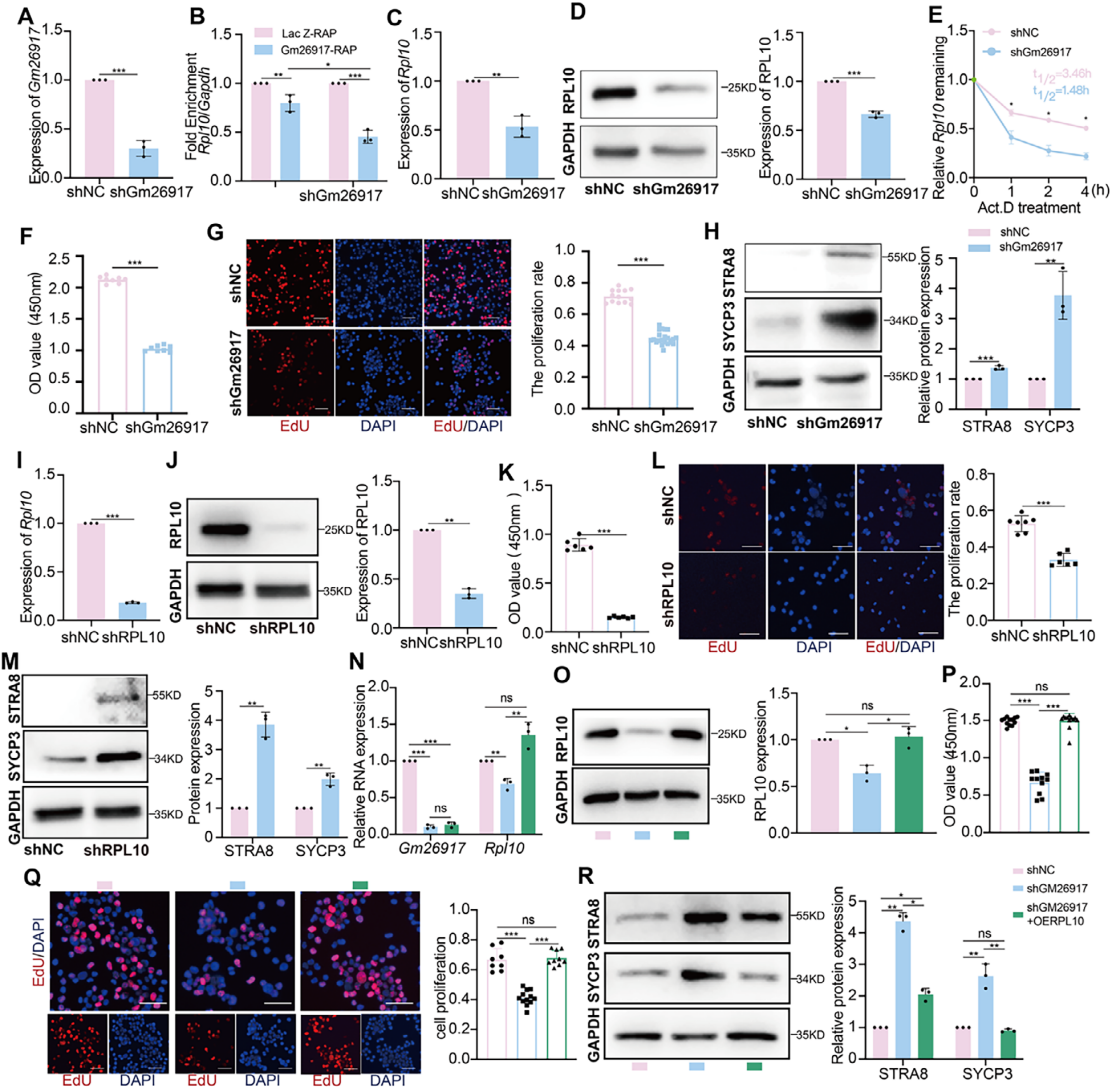
The disruption of *Gm26917*‐*Rpl10* interaction leads to impaired FGSC maintenance. (A) *Gm26917* knockdown efficiency was assessed by RT‐qPCR. (B) RAP‐qPCR showing *Rpl10* binding to *Gm26917* probes in shNC and shGm26917. (C and D) RPL10 expression showed significant decrease after shGm26917, detected by RT‐qPCR (C) and western blotting (D). (E) *Rpl10* half‐life assay with DRB. (F and G) Impaired viability (CCK‐8: F) and proliferation (EdU: (G) after shGm26917. (H) Upregulated differentiation markers (STRA8/SYCP3). (I–M) shRPL10 phenocopies shGm26917 effects (RPL10 expression: I, J; viability/ proliferation: K, L; differentiation: (M). (N–R) RPL10 rescue experiments (RPL10 expression: N, O; viability/ proliferation: P, Q; differentiation: (R). Scale bars: 10 µm (C): 50 µm (others). ^*^ *p* < 0.05, ^**^ *p* < 0.01, ^***^ *p* < 0.001; ns: not significant. Data: mean ± SD (n = 3 biological replicates).

This regulatory cascade was further confirmed by *Gm26917* knockdown (shGm26917; Figure S6A; Figure 6A), which diminished *Rpl10* binding (Figure 6B), reduced RPL10 expression levels (Figure 6C, D), and accelerated *Rpl10* mRNA decay (3.46 to 1.48 h; Figure 6E). These results establish an ac4C‐dependent mechanism whereby *Gm26917* safeguards *Rpl10* mRNA stability through direct RNA‐RNA interaction. Given the phenotypic parallels between shNAT10 and shGm26917, we probed *Gm26917*'s role in FGSC development. shGm26917 impaired FGSC viability and proliferation (Figure 6F, G), upregulated meiotic markers (STRA8 and SYCP3; Figure 6H), and disrupted cell cycle progression (reduced G0/G1, increased S‐phase;  Figure S6B), via dysregulation of cyclins (↑CDK4/cyclin D1; ↓cyclin A2/B1; Figure S6C). Moreover, shGm26917 elevated apoptotic (Figure S6D), with increased cleaved PARP, cleaved caspase‐3, and BAX, and reduced BCL‐2 (Figure S6E).

Strikingly, shRPL10 (Figure S6F and Figure 6I, J) phenocopied shGm26917, suppressing cell viability and proliferation (Figure 6K,L), promoting cell differentiation (Figure 6M), and altering cell cycle/apoptosis markers (Figure S6G–J). Critically, *Rpl10* overexpression (OERPL10; Figure S6K) rescued shGm26917‐induced defects, restoring RPL10 levels (Figure 6N, O), proliferation (Figure 6P, Q), and normalizing differentiation, cell cycle, and apoptotic profiles (Figure 6R and Figure S6L–N). These data delineate a novel epitranscriptomic circuitry wherein NAT10‐mediated ac4C modification endows *Gm26917* with the capacity to stabilize *Rpl10* mRNA, thereby orchestrating FGSC fate decisions. The identical phenotypic spectra observed in shNAT10, shGm26917, and shRPL10 conditions underscore the biological coherence of this regulatory axis.

### 2.6 Diminished lncRNA *Gm26917*‐*Rpl10* mRNA Interaction Impairs mRNA Translation Efficiency in FGSCs

**FIGURE 7:**
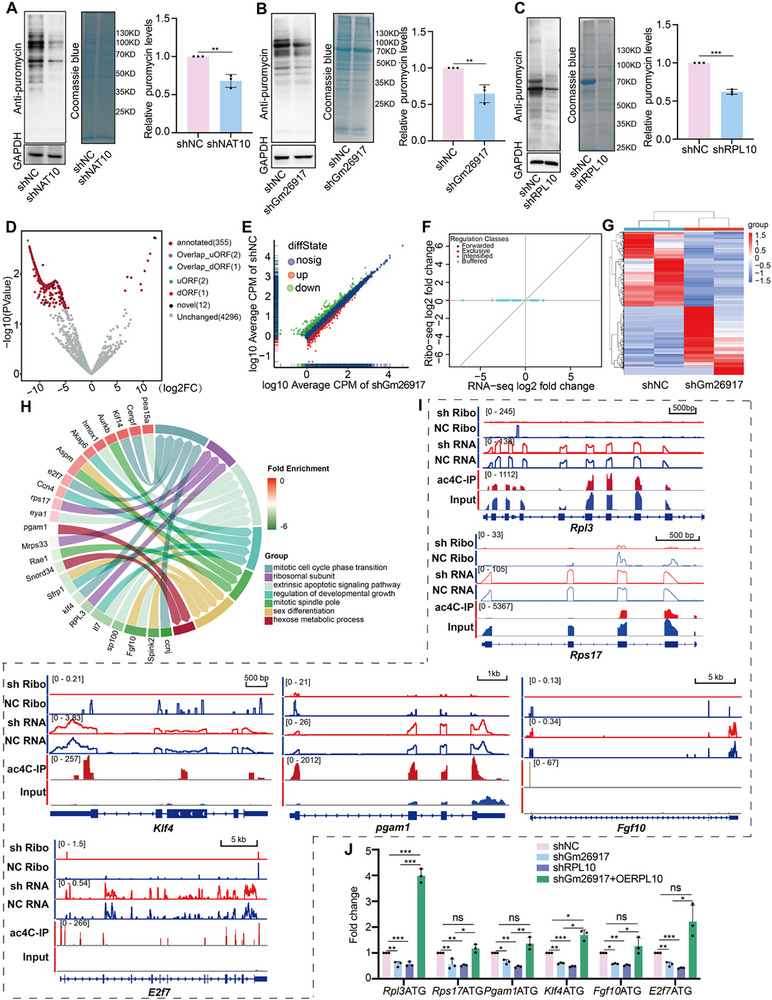
*Gm26917* knockdown impairs mRNA translation efficiency. (A–C) Global translation changes assessed by puromycin incorporation following shNAT10 (A), shGm26917 (B), or shRPL10 (C) treatment. Coomassie blue staining and GAPDH serve as loading controls (bottom: quantification of puromycin signal intensity). (D) ORF count distribution in shNC vs. shGm26917 FGSCs. (E) Differential gene expression profile (up‐/down‐regulated genes). (F) Scatter plot of deltaTE gene classification. (G) Heatmap of differentially translated genes (TEs). (H) Functional enrichment analysis of TE‐altered genes. (I) IGV tracks showing Ribo‐seq, RNA‐seq, and acRIP‐seq profiles for representative genes (*Rpl3*, *Rps17*, *Klf4*, *Pgam1*, *Fgf10*, and *E2f7*). (J) Translation initiation efficiency (ATG site) for selected genes across treatment conditions (shNC, shGm26917, shRPL10, and shGm26917+ OERPL10 FGSCs). ^*^ *p* < 0.05, ^**^ *p* < 0.01, ^***^ *p* < 0.001 (Student's t‐test; mean ± SD; Ribo‐seq: n = 2 biological replicates; other results: n = 3 biological replicates). Significance threshold: FC ≥ 1.5, p ≤ 0.05.

As an X chromosome‐encoded ribosomal assembly factor, RPL10 is essential for ribosome biogenesis. Puromycin incorporation assays demonstrated that shNAT10, shGm26917, or shRPL10 significantly impaired global translation efficiency (TE) in FGSC (Figure 7A–C), mechanistically linking the *Gm26917*‐*Rpl10* interaction to translation regulation. We found that reduced ac4C modification destabilized *Gm26917*, weakening its binding to *Rpl10* mRNA, ultimately decreasing RPL10 protein levels and disrupting FGSC development.

To elucidate the translational regulatory network governed by *Gm26917*, we performed Ribosome profiling and sequencing (Ribo‐seq) and RNA‐seq of *Gm26917* knockdown FGSCs. Principal component analysis (PCA) confirmed high reproducibility across biological replicates (Figure S7A), and fragment length distribution validated sequencing quality (Figure S7B). Using RiboCode, we identified and mapped active open reading frames (ORFs) by detecting P‐sites enriched at start codons (Figure S7C). Systematic classification of ORFs across all samples (Figure S7D, E) revealed 10 upregulated and 363 downregulated ORFs upon *Gm26917* depletion (Figure 7D).

Parallel RNA‐seq analysis (Figure S7F) and identified 521 upregulated and 611 downregulated genes (Figure 7E). Integrating of Ribo‐seq and RNA‐seq data via differential TE analysis uncovered 401 genes with increased TE and 381 with decreased TE (Figure 7F, G). Reactome (Figure S7G) and GO enrichment analyses (Figure S7H) highlighted significant enrichment of ribosome assembly‐related processes, consistent with RPL10 depletion. Strikingly, beyond ribosomal genes (*Rpl3*, *Rps17*), *Gm26917* knockdown selectively suppressed TE of key regulators governing differentiation (*Fgf10*), cell cycle progression (*Klf4*, *E2F7*), apoptosis (*Fgf10*), and metabolic process (*Pgam1*) (Figure 7H). Ribo‐seq confirmed reduced ribosome occupancy on these transcripts (Figure 7I), further validated by translational rate assays (Figure 7J). Critically, RPL10 overexpression (OERPL10) in shGm26917 FGSCs rescued TE defects, reinforcing the functional hierarchy of this axis. Our findings establish that ac4C‐modified *Gm26917* sustains *Rpl10* mRNA interaction to orchestrate translation efficiency of a network of genes critical for FGSC maintenance. Disruption of this epitranscriptomic checkpoint phenocopies RPL10 depletion, underscoring its central role in germline stem cell homeostasis.

### 2.7 EEF1A1 mediates the *Gm26917*‐*Rpl10* Interaction to Regulate mRNA Translational Efficiency

**FIGURE 8:**
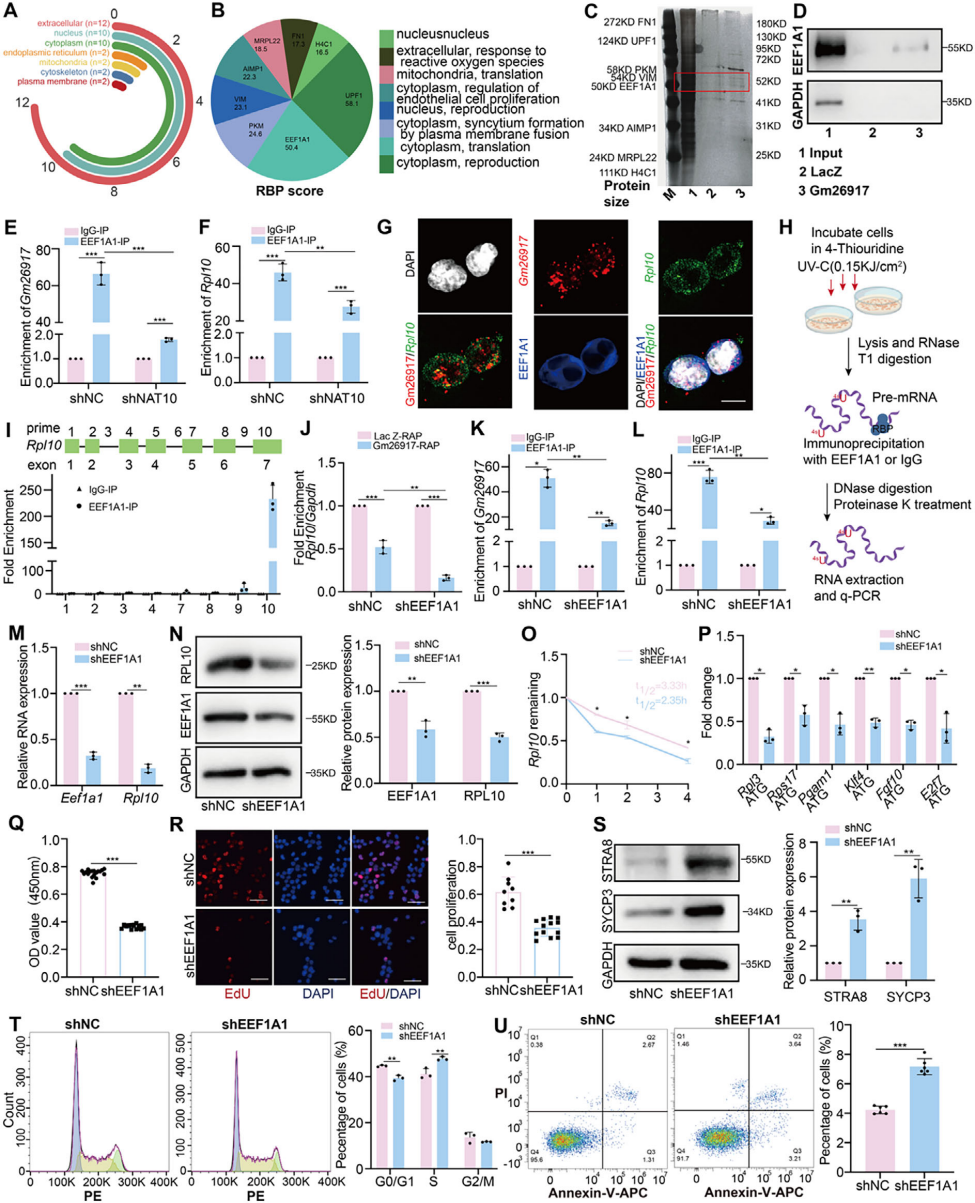
EEF1A1 regulates FGSC development through the *Gm26917*‐*Rpl10* axis. (A) Subcellular localization profile of candidate proteins. (B) RBP prediction scores, cellular localization and functional annotations of candidate proteins. (C and D) EEF1A1‐*Gm26917* interaction validated by RAP‐silver staining (C) and western blotting (D) (LacZ: negative control probes, Gm26917: *Gm26917* probes). (E and F) EEF1A1 RIP‐qPCR detected the affinity of EEF1A1 to *Gm26917* (E) and *Rpl10* (F) following *Nat10* knockdown. (G) FISH‐IF was used to analyze the co‐localization of EEF1A1 with *Gm26917* and *Rpl10*. Scale bars: 10 µm. (H) Schematic diagrams of the CLIP‐qPCR. (I) CLIP assays were performed to confrmed the interaction between EEF1A1 and the region of *Rpl10*. (J) RAP‐qPCR showing reduced *Rpl10* association with *Gm26917* probes upon *E* *ef1a1* knockdown. (K and L) EEF1A1 RIP‐qPCR confirming decreased binding to *Gm26917* (K) and *Rpl10* (L). M and N) RPL10 expression analysis by qRT‐PCR (M) and western blotting (N). (O) *Rpl10* mRNA stability assay using DRB treatment. (P) The translation rate at translation initiation site (referred to as ATG) of *Rpl3*, *Rps17*, *Pgam1*, *Klf4*, *Rps17*, *E2f7*, and *Fgf10* in shNC and shEEF1A1. (Q and R) CCK8 detected cell viability (Q) and EdU detected cell proliferation (R) upon *Eef1a1* knockdown. (S) Upregulation of meiotic markers (STRA8/SYCP3). (T and U) Cell cycle distribution (T) and apoptosis rate (U) by flow cytometry. Scale bars: 50 µm. ^*^ *p* < 0.05, ^**^ *p* < 0.01, ^***^ *p* < 0.001 (Student's t‐test; mean ± SD; n = 3).

To identify RNA‐binding proteins (RBPs) mediating the *Gm26917*‐*Rpl10* interaction, we performed RNA antisense purification followed by mass spectrometry (RAP‐MS) [31] (Figure 5E). Proteomic profiling identified 60 candidate proteins (Figure S8A), with molecular weights predominantly between 30–60 kDa (Figure S8B) and displayed the peptides number of per protein (Figure S8C). The integrated protein‐protein interaction (PPI) network analysis is shown in Figure S8D. We found 10 proteins subcellular localization in cytoplasm (Figure 8A). Using the RBP2GO database (https://rbp2go.dkfz.de/), we systematically evaluated their RBP potential. We analyzed the proteins with top‐ranked RBP scores using MS data and revealed their subcellular localization patterns and physiological functions (Figure 8B). Taken together, EEF1A1 emerged as the top candidate, supported by silver staining revealing a specific ∼50 kDa band in *Gm26917* pull‐downs (vs. lacZ control; Figure 8C), and western blotting confirmed EEF1A1 enrichment (Figure 8D). EEF1A1 RIP‐qPCR demonstrated EEF1A1 binds both *Gm26917* and *Rpl10*, with binding attenuated upon shNAT10 (Figure 8E, F), and cytoplasmic co‐localization was confirmed by RNA FISH‐immunofluorescence (Figure 8G and Figure S8F). In order to more precisely map the interaction sites of EEF1A1 with *Rpl10*, we performed CLIP‐qPCR using specifically designed primers (Figure 8H). The results indicated that EEF1A1 appeared to bind in exon7 of *Rpl10* (Figure 8I).

For functional validation, *Eef1a1* knockdown (shEEF1A1; Figure S8G, H) significantly reduced *Gm26917*‐*Rpl10* interaction (Figure 8J) and EEF1A1 binding enrichment with *Gm26917* and *Rpl10* (Figure 8K, L), decreased RPL10 expression levels (Figure 8M, N), and shortened *Rpl10* mRNA half‐life (3.33 to 2.35 h; Figure 8O). Notably, knockdown of *Eef1a1* recapitulated the translational efficiency reduction seen for specific mRNAs in *Gm26917*‐deficient FGSCs (Figure 8P). Phenotypically, EEF1A1 depletion recapitulated *Gm26917*/*Rpl10* knockdown effects, with impaired FGSC viability and proliferation (Figure 8Q, R), enhanced FGSC differentiation (Figure 8S), S‐phase cell cycle arrest (Figure 8T), and increased FGSC apoptosis (Figure 8U).

## 3 Discussion

FGSCs represent a crucial cellular reservoir for female fertility maintenance and regenerative therapies. Our study establishes ac4C modification as a master regulator of FGSC fate through a novel lncRNA‐mediated mechanism. Three key findings emerge: (1) ac4C modification is dynamically regulated during FGSC differentiation and essential for their maintenance; (2) The ac4C‐modified lncRNA *Gm26917* scaffolds a translational regulatory network by stabilizing *Rpl10* mRNA; (3) This axis requires the RBP EEF1A1 as a molecular bridge, revealing an unprecedented epitranscriptomic regulatory paradigm in stem cell biology.

ac4C modification governs FGSC maintenance. ac4C, m5C, and m6A are all involved in regulating RNA stability and translation efficiency. Unlike other RNA modifications, ac4C exhibits a unique position‐dependent dual regulatory role, promoting translation and mRNA stability within coding regions while inhibiting translation initiation at 5′ UTRs, whereas no clear regional preference has been observed in lncRNAs [32, 33, 34, 35]. In this study, the differentiation‐coupled decline in ac4C levels, recapitulated by NAT10 inhibition, and the concomitant bidirectional modulation of ac4C on genes (decreases with downregulation and increases with upregulation) point to a complex regulatory network governing ac4C dynamics in FGSC homeostasis. But the dynamics of ac4C on other RNA types beyond mRNA and lncRNA, as well as the regulatory machinery (such as other writers, erasers, and readers) governing these modifications, warrant future investigation. ac4C‐seq analysis revealed conserved modification patterns consistent with prior reports in other systems [32, 36, 37], but with distinct functional consequences in FGSCs. Moreover, the mechanism underlying the RE‐induced reduction of NAT10 protein levels in FGSCs requires further investigation. Genetic ablation (DDX4creERT2) and pharmacological inhibition experiment conclusively demonstrated that ac4C depletion triggers FGSC differentiation while impairing proliferation and viability, establishing NAT10 as a guardian of the undifferentiated state.

The ac4C‐*Gm26917*‐*Rpl10* regulatory axis plays a critical role in the regulation of FGSC fate by stabilizing the *Rpl10* mRNA through the action of the ac4C‐modified lncRNA *Gm26917*. Beyond its canonical roles in mRNA stability/translation [1, 6, 38], we discover that ac4C orchestrates functional RNA interactomes. RIC‐seq analysis identifies *Gm26917* as a hub lncRNA with numerous interactions, among which the *Gm26917*‐*Rpl10* pair emerged as particularly consequential. Mechanistically, ac4C modification stabilizes *Gm26917* (6.78 to 3.4 h upon *Nat10* knockdown). *Gm26917* in turn stabilizes *Rpl10* mRNA (3.46 to 1.48 h upon *Gm26917* knockdown), and boosts mRNA translation efficiency. This occurs despite *Rpl10* mRNA lacking detectable ac4C sites, revealing a trans‐stabilization mechanism. The phenotypic convergence of shNAT10, shGm26917, and shRPL10 conditions‐rescued by RPL10 overexpression—establishes this axis as biologically coherent and functionally essential.

EEF1A1 mediates the lncRNA‐mRNA interaction. Proteomic and biochemical identified EEF1A1 (RBP) as the physical bridge between *Gm26917* and *Rpl10*. This interaction requires ac4C modification (reduced binding in shNAT10). Also, the interaction is EEF1A1‐dependent (abrogated by *E* *ef1a1* knockdown), and controls *Rpl10* mRNA stability and translation. Notably, translationally repressed targets in shGm26917 cells showed concordant TE reduction in shEEF1A1 cells, demonstrating functional conservation of this regulatory node.

**FIGURE 9:**
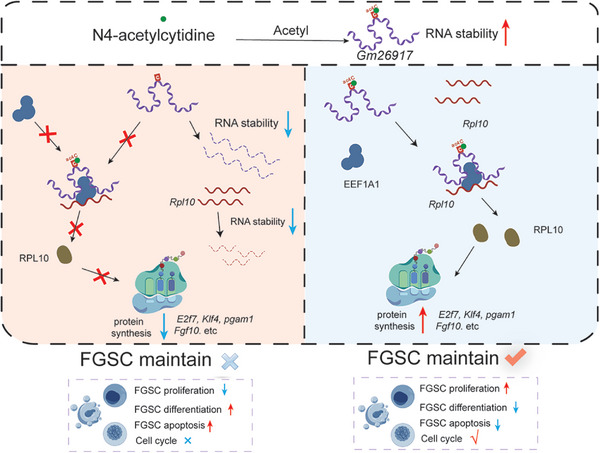
Working model: ac4C‐dependent translational regulation of FGSC fate. Schematic illustrating the ac4C‐*Gm26917*‐EEF1A1‐*Rpl10* regulatory axis: NAT10‐mediated ac4C modification stabilizes *Gm26917*; EEF1A1‐mediated *Gm26917* recruits *Rpl10* mRNA; Coordinated regulation maintains translational homeostasis essential for FGSC self‐renewal. Schematic diagrams were created with BioGDP.com.

This study reveals important biological and therapeutic implications. The ac4C‐*Gm26917*‐EEF1A1‐*Rpl10* axis represents a previously unrecognized mechanism coordinating ribosome biogenesis with stem cell fate decisions (Figure 9). Several lines of evidence underscore its physiological relevance. 1. Disease relevance: The observed phenotypes mirror ribosomopathies caused by RPL10 mutations in T‐cell acute lymphoblastic leukemia (T‐ALL), where RPL10 Arg98Ser disrupts ribosome biogenesis and drives oncogenic transformation [39, 40]. This parallel suggests conserved molecular mechanisms between germ cell development and hematopoietic malignancies; 2. Developmental conservation: The axis shows remarkable functional parallels with meiotic sex chromosome inactivation (MSCI) during spermatogenesis, where the testis‐specific paralog *Rpl10L* compensates for transcriptional silencing of *Rpl10* [41]. This conservation across germ cell lineages highlights its fundamental role in gametogenesis; 3. Regulatory breadth: The network coordinately controls master regulators of germ cell biology, including *Fgf10*, *Klf4*, *Pgam1*, and *E2f7*, demonstrating its pleiotropic effects on FGSC fate determination. The selective ac4C modification of regulatory RNAs (*Gm26917*) but not structural ribosomal components (*Rpl3*, *Rps17*) suggests a sophisticated epitranscriptomic code distinguishing regulatory vs. housekeeping transcripts in FGSCs.

Future directions emerge from this study. While we establish ac4C's role in lncRNA stability, the structural consequences of this modification remain to be elucidated. Potential mechanisms include altered RNA secondary structure, modulation of RBP binding landscapes, and changes in subcellular localization. Furthermore, the therapeutic potential of targeting this axis ‐either through NAT10 inhibition or EEF1A1 modulation—warrants investigation in fertility preservation and regenerative contexts.

In conclusion, our work reveals that ac4C modification coordinates gene expression through both direct RNA stabilization and three‐dimensional organization of lncRNA‐mRNA interactomes. By identifying ac4C‐*Gm26917*‐EEF1A1‐*Rpl10* axis as a central regulator of FGSC fate, we expand the functional repertoire of epitranscriptomic modifications and provide new molecular targets for FGSC maintenance and ovarian repair.

## 4 Experimental Section

### 4.1 Mouse Models

The floxed *Nat10* (*Nat10* flox/flox) alleles and DDX4CreERT2 transgenic mice were provided by Prof. Jianqiang Bao's laboratory [42]. The germ cell‐specific *Nat10*‐deficient mice (*Nat10*‐DcKO) were generated by crossing *Nat10* flox/flox mice with DDX4‐CreERT2 transgenic mice. Successful recombination was confirmed by RT‐PCR genotyping. To induce NAT10 deletion, DDX4+/cre, NAT10flox/flox mice received intraperitoneal tamoxifen injections (10540‐29‐1, Mce) dissolved in corn oil (8001‐30‐7, Mce) for four consecutive days.

All mice were maintained on a C57BL/6J background. Animal experiments were approved by the Institutional Animal Care and Use Committee of the University of Science and Technology of China (USTC; ethical approval: A2019118).

### 4.2 Culture and Differentiation of FGSCs

FGSCs were cultured and differentiated as previously described [28, 30]. For differentiation, FGSCs were co‐cultured with granulosa cells (GCs) in MEM‐α medium (12000063, Gibco) supplemented with 5% fetal bovine serum (FBS; Front), 10 ng/mL bFGF (PeproTech), 0.1 µM retinoic acid (RA; R2625, Sigma), 10 ng/mL bone morphogenic protein (BMP4; PeproTech), 1 mM nonessential amino acids (NEAA; 11140050, Gibco), 1 mM sodium pyruvate (11360070, Gibco), 2 mM L‐glutamine (25030081, Gibco) and 0.1 mM β‐mercaptoethanol (444203, Sigma). Cells were maintained at 37°C with 5% CO_2_, and the medium was refreshed every 48 h.

### 4.3 RNA Isolation, RT‒PCR, and qRT‒PCR

Total RNA was extracted using TRIzol reagent (DP424, Tiangen), reverse‐transcribed with cDNA Synthesis SuperMix (11141ES60, Yeasen). The RT‐PCR was performed with 2x Hieff PCR Master Mix (10106ES03, Yeasen) and detected by Gel electrophoresis. The expression of the target genes was measured by Universal Blue qPCR SYBR Green Master Mix (11184ES03, Yeasen). *G* *apdh* served as the internal control. The primer sequences are shown in Table S1.

### 4.4 Western Blotting

Proteins were lysed in RIPA buffer (P0013B, beyotime) with 1× protease inhibitor cocktail (P1005, beyotime), quantified via BCA protein quantification kit (20200ES76, Yeasen), and desaturated in 5× loading buffer (C508320, Sangon Biotech). Samples were separated by sodium dodecyl sulfate‐polyacrylamide gel electrophoresis (SDS‐PAGE) and transferred onto polyvinylidene difluoride (PVDF) membranes. Membranes were blocked with non‐fat milk in TBST, and probed with primary and secondary antibodies. Signals were detected using Super ECL Reagent (36208ES60, Yeasen) on a FUSION FX imager (VILBER, France). The details of antibodies are listed in Table S2.

### 4.5 RNA Dot Blotting for ac4C

The ac4C dot blottingting was conducted to detect the ac4C level of total RNA and mRNA as previously described [43]. Total RNA or poly(A)‐enriched mRNA (isolated using Dynabeads mRNA Purification Kit, 61006, Invitrogen) was denatured with denaturation solution (20X SSC solution: RNA = 1:1) at 95°C for 5 min, spotted onto nitrocellulose membrane, and UV‐crosslinked (UV 254 nm, 6 min). Membranes were stained with methylene blue (MB, M4159, Sigma) for 5 min, washed with 75% ethanol, PBST buffer (containing 0.02% Tween 20), and blocked. Then membranes were probed with anti‐ac4C antibody (ab252215, Abcam; 1:1000) followed by HRP‐conjugated secondary antibody (sa00001‐2, Proteintech; 1:2000). Signals were visualised using a FUSION FX imager (VILBER, France).

### 4.6 Immunofluorescence

Cells were washed with PBS and fixed with 4% paraformaldehyde (PFA), followed by treatment with 0.5% Triton X‐100 (T9284, Sigma) and blocked with 10% goat serum after washing. Then, the cells were incubated sequentially with primary antibodies and then Alexa Fluor 488/or Alexa Fluor 594‐conjugated secondary antibodies. The nucleus was stained with 40,6‐diamidino‐2‐phenylindole (DAPI, C1002, Beyotime), and DMI3000B fluorescence microscope (Leica) was used to obtain images.

### 4.7 acRIP‐seq

FGSCs and Dif‐FGCs were subjected to ac4C RNA immunoprecipitation and sequencing (acRIP‐seq) by CloudSeq Inc. (Shanghai, China) as previously described. [1, 32, 43] In short, total RNA was extracted, followed by ribosomal RNA (rRNA) depletion using NEBNext rRNA Depletion Kit v2. For library preparation, RNA Fragmentation Reagents were used to fragment the RNA to ∼200 nt. Then, the RNA fragments were incubated with the protein A beads/ ac4C antibody complexes for 4 h at 4°C. Eluted the ac4C‐enriched RNA fragments off the beads by proteinase K digestion, and purified the RNA fragments by phenol‐chloroform. Subsequently, the RNA fragments were used for RNA library construction. RNA Library quality was detected by Agilent 2100 bioanalyzer, and Library sequencing was performed on the NovaSeq platform (Illumina). Raw sequence data and processed data were submitted to the GEO and are accessible through the accession number GEO: GSE298164.

### 4.8 Lentiviral Infection and Screen of FGSCs

Lentivirus infection was performed according to the manufacturer's protocol. FGSCs were infected with lentiviruses encoding shRNAs against *Nat10* (shNAT10, Obio technology, China), *Gm26917* (shGm26917, Obio technology, China), or *Rpl10* (shRPL10, Genomeditech bio‐technology, China), or overexpressing *Nat10* (OENAT10, HANbio, China). Briefly, cells were infected with lentivirus at 30–40%, and infected cells were selected using 1–5 µg/mL puromycin (ST551, Beyotime) or 1–5 µg/mL blasticidin S (B796830, Macklin), depending on the resistance genes encoded by the lentiviral vector. The oligonucleotides of the siRNA sequence are shown in Table S3.

### 4.9 CCK‐8 and EdU Labeling Assay

Cell counting Kit 8 (CCK‐8) assay (C0037, beyotime) was used to detecte cell viability, and the 5‐Ethynyl‐2’‐Deoxyuridine (EdU) assay (C10310‐1, RiboBio) was performed to evaluate cell proliferation according to the manufacturer's instructions.

### 4.10 Flow Cytometry Analysis

Cell cycle and apoptosis were analyzed by flow cytometry (BD FACSCalibur machine, BD Biosciences) using Propidium staining (C1052, beyotime) and annexin V APC/propidium iodide (PI) (88‐8007‐74, Thermo Fisher) staining, respectively.

### 4.11 hematoxylin‐eosin Staining

The ovaries were fixation with 4% paraformaldehyde and embedded in paraffin [44]. Ovarian specimens were processed through paraffin embedding and serially sectioned at 5 µm thickness. Following conventional hematoxylin‐eosin (H&E) staining, follicular enumeration was conducted per established histological criteria [45]. Besides, the paraffin sections were stained by immunofluorescence after antigen repair.

### 4.12 Immunohistochemistry

The mice were injected with 200 µg EdU (A10044, Thermo Fisher) after being injected with tamoxifen for four consecutive days. The ovaries were flash frozen with OCT (14020108926, Leica). The frozen section was circled with an immunohistochemical pen (J417FC0551, BBI Life Sciences). Then EdU staining was accomplished by kit (C10339, Thermo Fisher) according to the manufacturer's instructions, following incubated with DDX4‐antibody, Alexa Fluor 488‐conjugated secondary antibodies (RGAR002, Proteintech), and the nucleus was stained with DAPI. The images were photographed under a DMI3000B fluorescence microscope (Leica).

### 4.13 Germ Cell Enrichment from Mouse Ovaries

FGSCs and oocytes were released from isolated ovaries of WT and KONAT10 mice by mincing and enzymatic digestion. Following a 3 h differential adhesion, non‐adherent cells were collected, and germ cells were purified with DDX4 magnetic beads for total RNA extraction. The RNA isolated from germ cells of mouse ovaries was used for dot blotting and RT‐qPCR analysis.

### 4.14 Long RNA Sequencing (LongRNA‐seq)

Total RNA extracted with TRIzol was used to prepare rRNA‐depleted libraries (Epi Mini LongRNA‐seq Kit, Guangzhou Epibiotek), which were sequenced on a NovaSeq 6000 (150 bp, paired‐end). Clean reads were aligned to the reference genome (HISAT2) and analyzed for differential expression (DESeq2). The data are available in the GEO database (accession number: GSE298163).

### 4.15 RIC‐seq

RNA in situ conformation sequencing (RIC‐seq) was completed by Guangzhou Epibiotek Co.,Ltd. Briefly, shNC and shNAT10 FGSCs were washed with PBS, crosslinked with PBS solution containing 1% formaldehyde, followed by quenching with glycine buffer. Subsequently, the cells were washed with PBS, scraped on ice, and centrifuged at 500×g for 10 min at 4°C. The cell pellet was snap‐frozen in liquid nitrogen and stored. The remaining steps were performed according to the manufacturer's protocol [17]. Samples were permeabilized with permeabilization buffer, 2 U/mL SUPERase In RNase inhibitor (AM2694, Thermo Fisher) on ice. RNAs subsequently were digested with MNase (1: 100,000 dilution, EN0181, Thermo Fisher) and treated with FastAP thermosensitive alkaline phosphatase (EF0651, Thermo Fisher) at 37°C to generate dephosphorylated 3’ overhangs. The RNA 3’end was labeled with pCp–biotin (20160, Thermo Fisher), followed by treatment with FastAP thermosensitive alkaline phosphatase and T4 PNK (EK0032, Thermo Fisher) at 37°C. After proximity ligation with T4 RNA ligase (EL0021, Thermo Fisher), ligated RNAs were extracted, and treated with DNase I. rRNA was removed xia the Ribo‐off rRNA depletion kit (N406‐01, Vazyme). Following rRNA depletion, the remaining RNA population underwent fragmentation, followed by C‐biotin affinity selection and subsequent strand‐oriented library preparation.

### 4.16 RIP‐qPCR

Both acRIP‐qPCR (to examine ac4C modification levels of target genes) and EEF1A1 RIP‐qPCR (to identify RNAs binding to EEF1A1) were performed using the immunoprecipitation kit (P0102, Geneseed Biotech, Guangzhou) according to the manufacturer's instructions. Briefly, RNA fragments were incubated with protein A/G magnetic beads pre‐bound to either 5 µg anti‐ac4C or anti‐EEF1A1 antibodies (4°C, overnight). The antibody‐binded RNA was eluted by elution buffer, then analyzed by qRT‒PCR. The primer sequences are shown in Table S1.

### 4.17 RNA Stability Assays

To evaluate RNA stability, cells were treated with 75 µM 5, 6‐dichloro‐1‐b‐D‐ribofuranosylbenzimidazole (DRB, C4798, APExBIO) for 0, 1, and 3 h. Total RNA was extracted, and the target mRNAs half‐life was determined by qRT‐PCR. The primer sequences are shown in Table S1.

### 4.18 Cross‐linking Immunoprecipitation and qPCR

Crosslinking‐immunoprecipitation (CLIP) was performed by CLIP kit (Bes3014, Bersin). Briefly, cells were pre‐treated with 4‐thiouridine and subsequently irradiated with 254 nm UV on ice for crosslink of protein to RNA. Cells were lysed and digested by RNase T1, and the protein‐RNA complexes were immunoprecipitated using EEF1A1 or IgG antibody. protein‐RNA complexes were incubated with Protein A/G beads, followed by DNase I treated, proteinase K digestion, and extracted. Purified RNAs were reverse‐transcribed into cDNA (M201, Yeasen). PCR reactions were set up and run using the specific forward primers and a universal tailed reverse primer. The enrichment values were normalized to the input sample and calculated using the 2^(−ΔΔCt) method. The primer sequences are shown in Table S1.

### 4.19 Puromycin Intake Assay

The puromycin intake assay was conducted according to the previous report [46, 47]. In short, the cells were incubated with puromycin (1 µg/mL; ST551, Beyotime) for 1 h at 37°C. The proteins were extracted with RIPA lysis buffer, and equal amounts of protein were used for western blotting. Proteins were incubated with puromycin antibodies (A21205, ABclonal).

### 4.20 Nucleotide‐resolution Method for Proling *Gm26917* ac4C Sites

Chemical reduction was performed by incubating RNA with 1 M sodium borohydride (NaBH_4_, 215511, Sigma) at RT for 20 min post‐HCl (1 M) activation. Reactions were quenched with Tris‐HCl (1 M, pH 8.0) before miRNeasy purification (217084, Qiagen). ac4C modification rates at *Gm26917* loci were determined by sequencing chromatogram analysis of RT‐PCR products [48, 49]. The primer sequences are shown in Table S1.

### 4.21 RAP‐qPCR and RAP‐MS

We designed a biotin‐labeled probe set of *Gm26917* (Bersinbio) and performed RNA antisense purification (RAP) [50] analysis using the RAP kit (Bes5103‐3, Bersinbio) to pull down the target RNA and the RNA‐binding proteins (RBPs) that interact with *Gm26917*. The target RNA or protein will be attached to the magnetic beads. We combined RAP with quantitative PCR (RAP‐qPCR) to detect the downstream target genes of *Gm26917*. Additionally, RBPs were analyzed using mass spectrometry (MS) to identify unknown interacting proteins, with the analysis conducted by PTM Bio. The proteins were detected using a silver staining kit (P0017S, Beyotime). The mass spectrometry data can be accessed in ProteomeXchange with the dataset identifier PXD063853. Information about *Gm26917* probes is listed in Table S4.

### 4.22 Single‐molecule Fluorescence In Situ Hybridization and Immunofluorescence

The co‐localization of lncRNA *Gm26917* and *Rpl10* mRNA was detected by the ViewRNA Cell Plus Assay Kit (GenePharma) according to the manufacturer's instructions. Briefly, cells grow on cover glass and are incubated with *Gm26917*‐28 Hybridization probe and *Rpl10*‐25 Hybridization probe, next stepwise incubation with amplification 28/25 probe, image 28 Cy5/25 Cy3 probe. Furthermore, to detect *Gm26917*‐*Rpl10* co‐localisation with target proteins, EEF1A1 or DDX4, antibody incubation was performed prior to probe hybridization, followed by fixation with PFA to preserve the antibody‐epitope binding. Following acquisition with a confocal microscope (Stellaris 5; Leica), images from each fluorescence channel were assigned appropriate pseudocolors using the LASX software. Fluorescence intensity was quantified using ImageJ, and Pearson's correlation coefficient, along with thresholded Manders’ coefficients (M1thr and M2thr) were calculated using the JACoP plugin [17, 51]. The information of *Gm26917* and *Rpl10* probes is listed in Table S5.

### 4.23 Ribo‐seq

Ribosome profiling and sequencing (Ribo‐seq) was completed by Geneseed Biotech Co.,Ltd (Guangzhou, China). In brief, the cells were treated with cycloheximide (CHX, 100 µg/mL; Sigma, USA) and washed with PBS (with CHX). Cells were scraped off and centrifuged to collect cell precipitates, which were then stored at ‐80°C. The remaining steps were carried out by a company according to previously described methods [43]. The ribo‐seq library was constructed by digesting cell lysates with RNase I, followed by the isolation of ribosome complexes through ultracentrifugation over a 1 M sucrose cushion. RNA was extracted using the miRNeasy Micro Kit, and approximately 28 nt ribosome‐protected fragments were purified by urea‐PAGE. A small RNA library was prepared through adapter ligation, reverse transcription, and PCR amplification, with final size selection performed using SPRI beads. Library quality was verified using a Bioanalyzer before equimolar pooling and sequencing on NovaSeq 6000. Raw sequence data and processed data were submitted to the GEO and are accessible through the accession number GEO: Ribo‐seq data, GSE304964, and the related RNA‐seq data, GSE304963.

### 4.24 Detection of Target mRNA Translation Rate

We measured the translation rates of target mRNAs at the initiation site using a modified “Targeted Profiling of RNA Translation” method [52, 53, 54]. Briefly, cells were treated with pre‐cooled 100 mg/mL cycloheximide (CHX) in PBS before lysis in a buffer containing 150 mM sodium chloride, 20 mM Tris (pH 7.4), 5 mM magnesium chloride, 1 mM DTT, 1% (v/v) Triton X‐100, and 1 mg/mL cycloheximide. The samples were then immediately frozen in liquid nitrogen. After thawing on ice, the lysates were scraped, centrifuged, and the supernatant was digested with RNase I at 4°C for 1 h. An RNase inhibitor was added to terminate the reaction. RNA was extracted using TRIzol reagent, reverse‐transcribed, and analyzed by qPCR. We assessed translation rates at initiation sites. The rate of *Gapdh* was used for comparative analysis. The primer sequences are listed in Table S1.

### 4.25 Quantification and Statistical Analysis

All experiments were performed at least in triplicate in this study. The gray of western blottings, Dot blottings, and IF intensity were quantified with ImageJ. Statistical significance of experiment results was analyzed by Graph‐Pad Prism 9 software (GraphPad, San Diego, CA, USA), Statistical significance was assessed using one‐way ANOVA or Student's *t*‐test, as appropriate. The details were described in the corresponding Figure legends. A p‐value of < 0.05 was considered statistically significant.

## Author Contributions

Ji Wu, Xinyue Li, and Xiaopeng Hu designed the experiments; Xinyue Li performed the experiments, analyzed and interpreted the data; Ji Wu, Xiaopeng Hu, and Xinyue Li wrote and modified the manuscript; Ji Wu concerned and supervised the study. All authors discussed results, reviewed and edited the manuscript.

## Conflicts of Interest

The authors declare no conflicts of interest.

## Supporting information




**Supporting File**: advs74863‐sup‐0001‐suppmat.docx.

**Supporting File**: advs74863‐sup‐0002‐blots.docx.

## Data Availability

The data that support the findings of this study are available from the corresponding author upon reasonable request.
