# Supplementary material for: N4‐acetylcytidine in LncRNA Gm26917 Promotes Translation in Female Germline Stem Cells by Recruiting Ribosomal Protein mRNA via EEF1A1
Source: Adv Sci (Weinh). 2026 Mar 17;13(30):e20059. doi: 10.1002/advs.202520059 (PMC13248794; doi:10.1002/advs.202520059)
Supplement: Supplementary file 1 — Supporting File: advs74863‐sup‐0001‐suppmat.docx. [file ADVS-13-e20059-s002.docx]

Supplementary Materials for

**N4-acetylcytidine in LncRNA *Gm26917* Promotes Translation in Female Germline Stem Cells by Recruiting Ribosomal Protein mRNA via EEF1A1**

Xinyue Li, Xiaopeng Hu*, Ji Wu*

Y. Li, P. Hu, J. Wu

Key Laboratory for the Genetics of Developmental and Neuropsychiatric Disorders (Ministry of Education), Bio‑X Institutes, Shanghai Jiao Tong University, Shanghai 200240, China.

E-mail: [jiwu@sjtu.edu.cn](mailto:jiwu@sjtu.edu.cn); [huxiaopeng2017@sjtu.edu.cn](mailto:huxiaopeng2017@sjtu.edu.cn)

J. Wu

Key Laboratory of Fertility Preservation and Maintenance of Ministry of Education, School of Basic Medical Sciences, Ningxia Medical University, Yinchuan 750004, China

J. Wu

Shanghai Key Laboratory of Reproductive Medicine, Shanghai 200025, China

**This PDF file includes:**

Figure S1 to S8

Tables S1 to S5


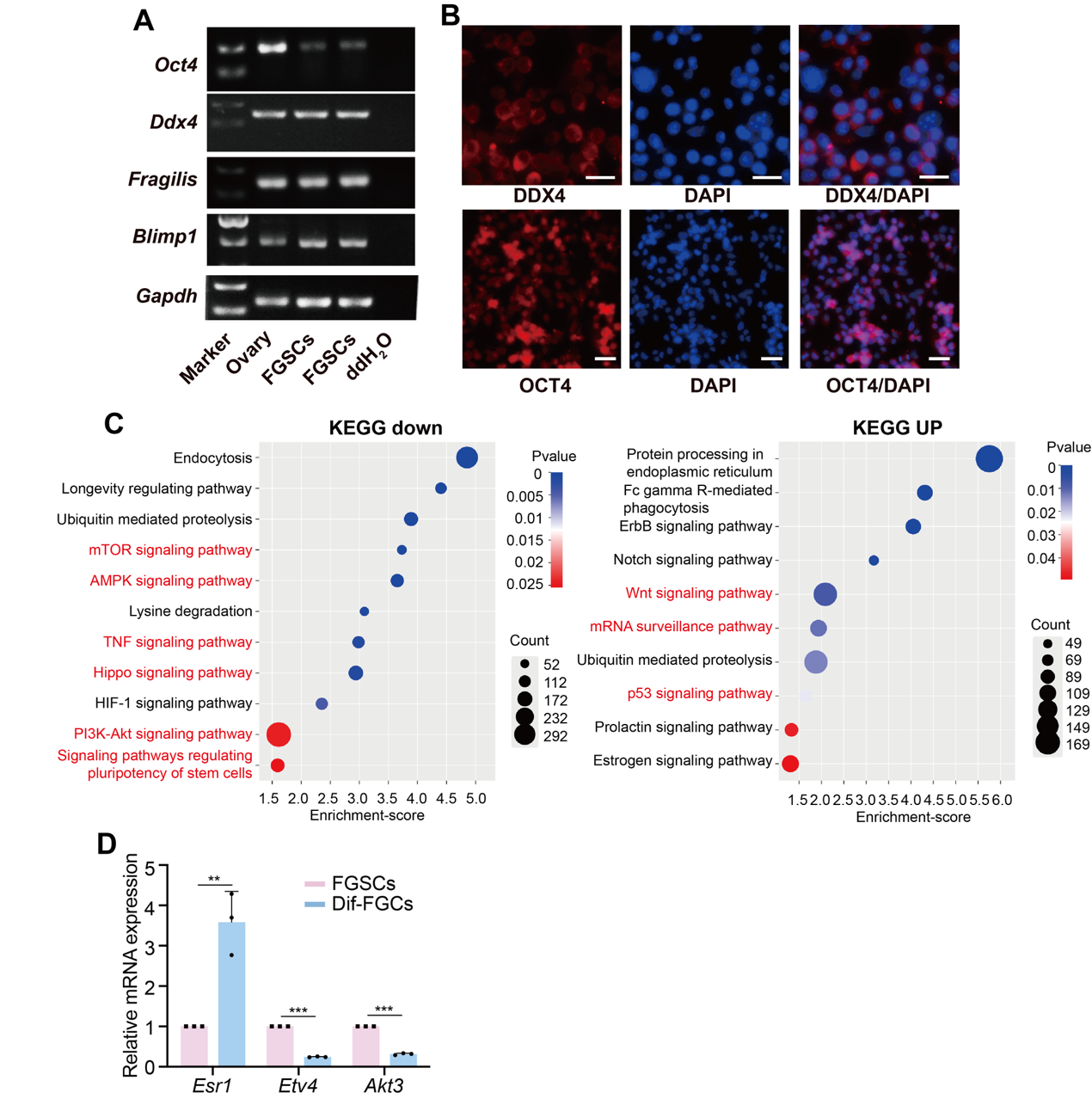


**Figure S1. Characterization of FGSCs in vitro and acRIP-seq data analysis. A)** RT-PCR analysis of germline markers (*Oct4*, *Ddx4*, *Fragilis*, and *Blimp1*) in FGSCs. **B)** Immunofluorescence of DDX4 and OCT4 in FGSCs. **C)** KEGG pathway enrichment of genes with differential ac4C modifications (downregulated: left; upregulated: right). **D)** RT-qPCR analysis the expression of *Esr1*, *Etv4*, and *Akt3* in FGSCs and Dif-FGCs. Scale bars: 50 μm. Data represent mean ± SD (n=3 biological replicates).

**
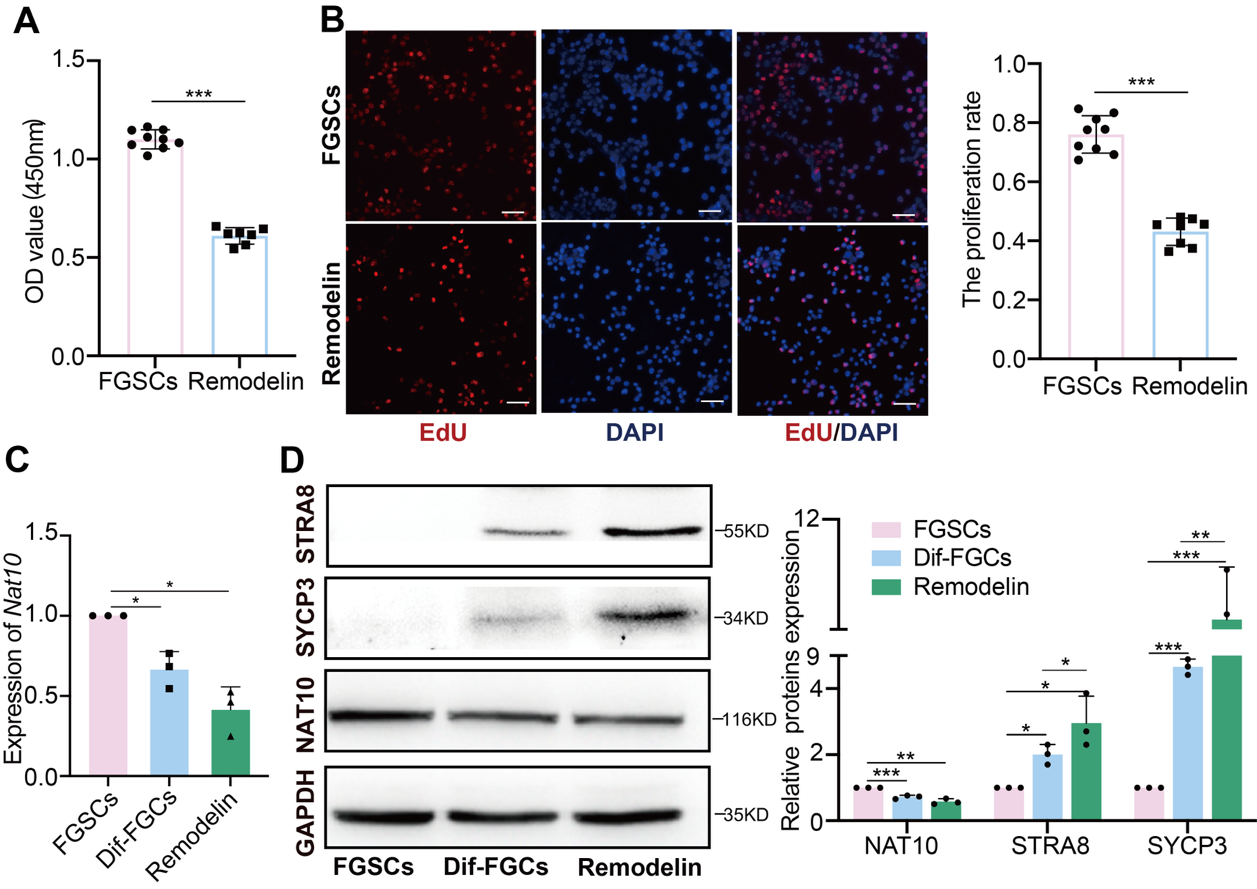
**

**Figure S2. Remodelin-induced FGSC differentiation in vitro. A and B)** Remodelin treatment suppressed FGSC viability (A) and proliferation (B). **C)** Detecting the expression of *Nat10* by RT-qPCR. **D)** Western blotting analysis of NAT10, STRA8, and SYCP3 expression across conditions, including FGSCs, Dif-FGCs, and Remodelin-treated FGSCs. Scale bars: 50 μm. *p<0.05, **p<0.01, ***p<0.001. Data represent mean ± SD (n=3).


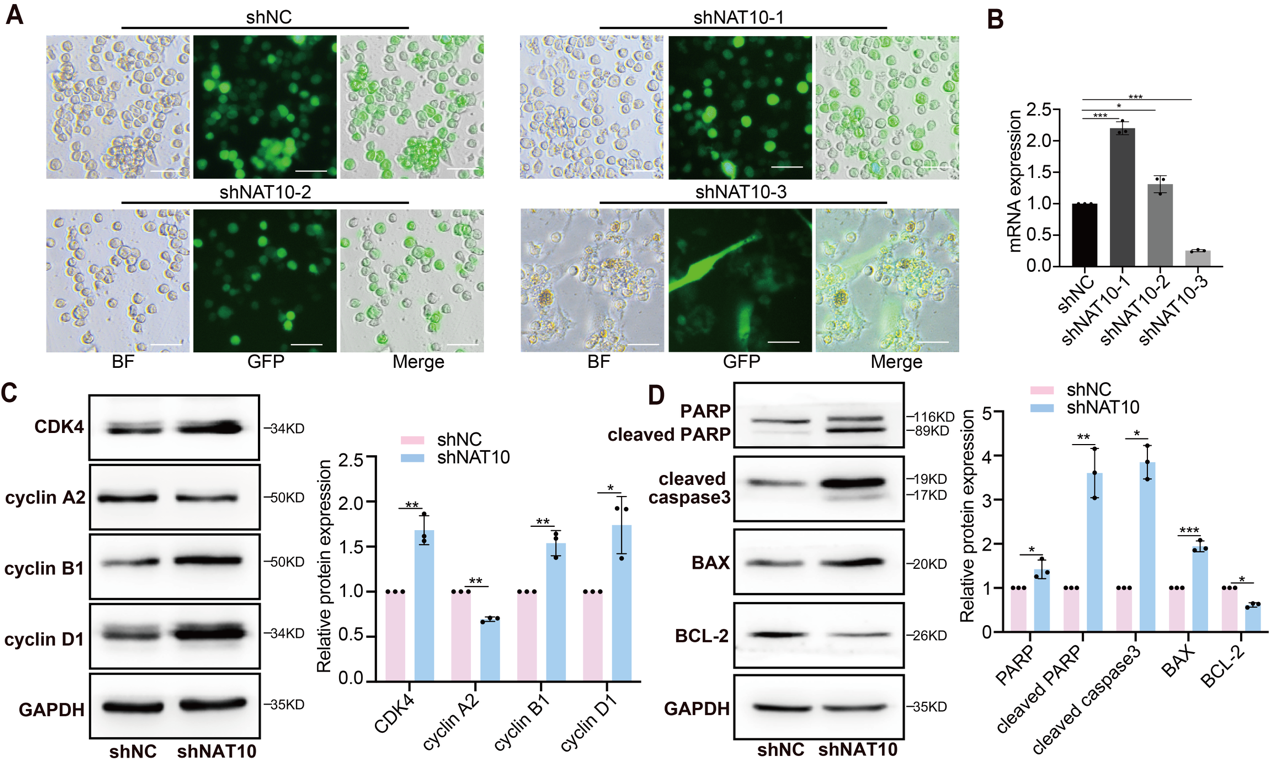


**Figure S3. Validation of shNAT10 lentiviral infection and analysis of cell cycle/apoptosis markers. A)** Morphological assessment of FGSCs following lentiviral infection with either control (shNC) or *Nat10*-targeting shRNA (shNAT10). **B)** Quantitative verification of *Nat10* knockdown efficiency by RT-qPCR. **C)** Western blotting analysis of key cell cycle regulatory proteins in shNC vs. shNAT10 FGSCs. **D)** Western blotting evaluation of apoptosis-related protein expression following *Nat10* knockdown. Scale bars: 50 μm. *p<0.05, **p<0.01, ***p<0.001. Data represent mean ± SD (n=3 biological replicates).


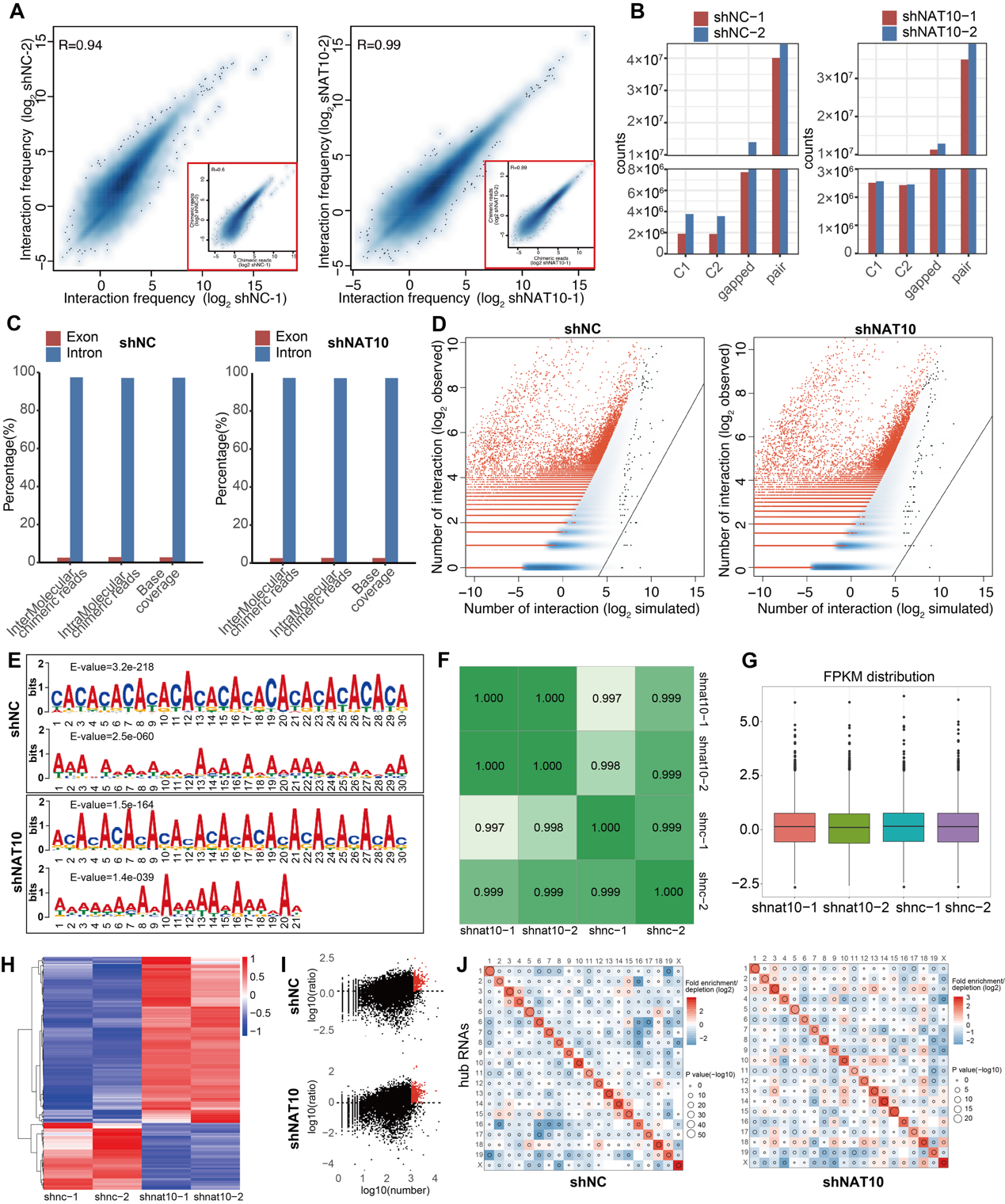


**Figure S4.** **Global RIC-seq and Long RNA-seq characteristics.** **A)** Replicate correlation scatter plots of RIC-seq interactions (red box: chimeric read per transcript). Both scatter plots display data normalized by RNA abundance. **B)** Read pair distribution in shNC/shNAT10 FGSCs (analyzed pairs shown), where pairs groups represent the final number used for subsequent analysis. **C)** The ratio of inter-, intramolecular chimeric reads in the intron and exon region in shNC and shNAT10. **D)** Distribution of high confidence interaction reads identified in duplicate samples. The observed pairwise interactions were compared with simulated random counts to identify high-confidence interactions (P≤ 0.05, red dots). **E)** RNA-RNA interaction sequence motifs. **F)** PCA analysis of shNC and shNAT10 FGSCs. **G)** FPKM distribution. **H)** Heatmap of DEGs between shNC and shNAT10 FGSCs. **I)** The display of Hub RNA in shNC and shNAT10 FGSCs. **J)** Hub RNAs from the same chromosome tend to interact with each other. Statistical criteria as in Figure 4.


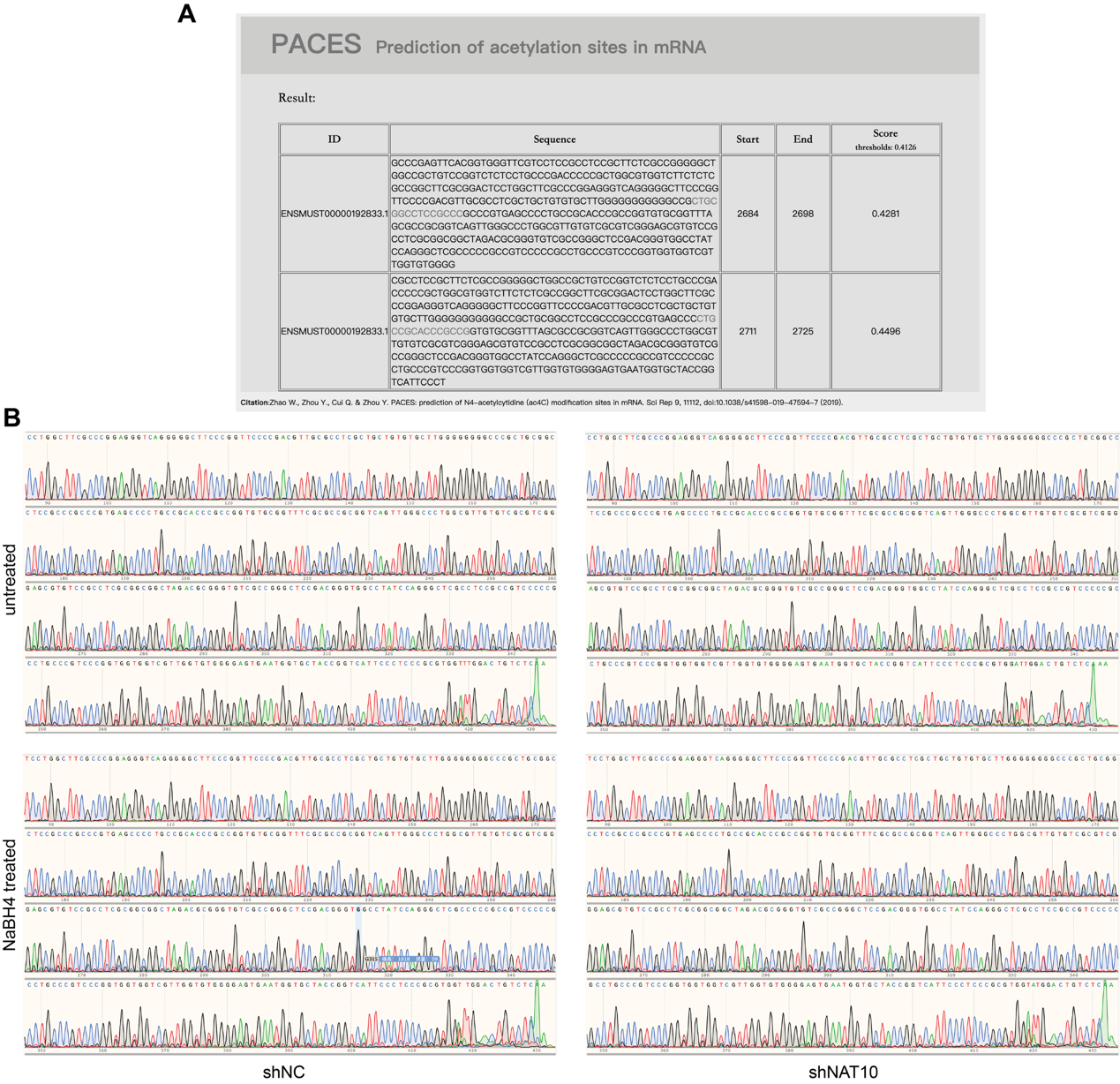


**Figure S5. Detection of ac4C modification on *Gm26917*.** **A) The prediction result of ac4C modification sites in *Gm26917*. B) Sanger DNA sequencing of *Gm26917* ac4C peak region after borohydride treatment of total RNA extracted from shNC and shNAT10 cells.**


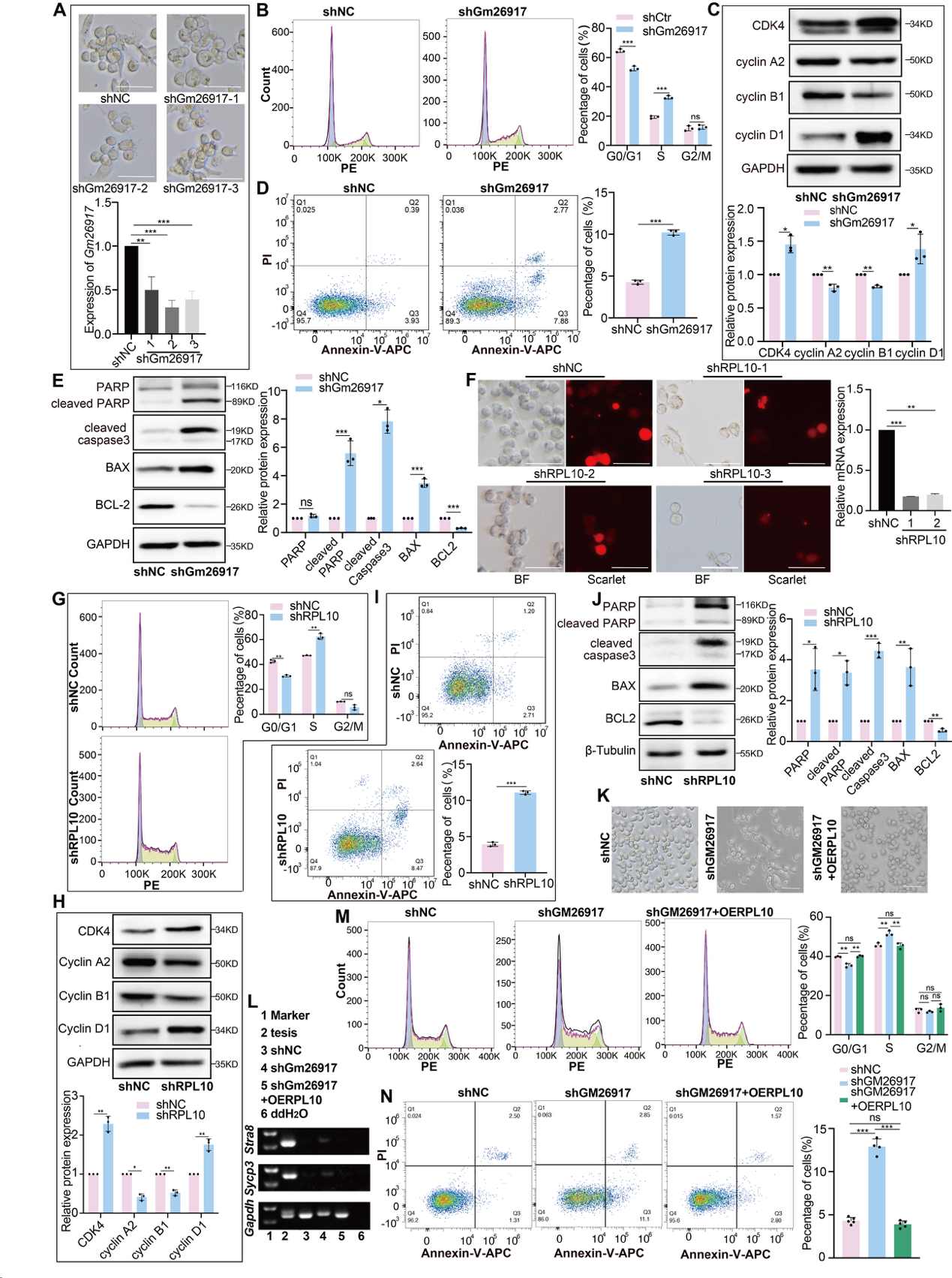


**Figure S6. *Gm26917*-*Rpl10* interaction regulates FGSC development.** **A)** Morphological analysis of FGSCs following lentiviral infection with shNC or three independent shGm26917 constructs (inset: RT-qPCR validation of knockdown efficiency). **B)** Cell cycle distribution (flow cytometry) in shNC vs. shGm26917 FGSCs. **C)** Western blotting analysis of cell cycle regulators. **D)** Apoptosis rates quantified by flow cytometry. **E)** Apoptosis-related protein expression profiles. **F)** FGSC morphology after shNC or shRPL10 lentiviral infection (Scarlet fluorescent tag). RT-qPCR validation of *Rpl10* knockdown. **G)** Cell cycle profiling post-shRPL10. **H)** Cell cycle regulator expression by Western blotting. **I)** shRPL10-induced apoptosis (flow cytometry). **J)** Apoptosis-related protein expression changes. **K)** Rescue experiment: FGSC morphology with shGm26917 ± RPL10 overexpression. **L)** Differentiation markers (*Stra8*/*Sycp3*) analyzed by RT-PCR. **M and N)** Cell cycle (M) and apoptosis (N) detection in rescue experiments. Scale bars: 50 μm. *p<0.05, **p<0.01, ***p<0.001 (mean ± SD; n=3 biological replicates).


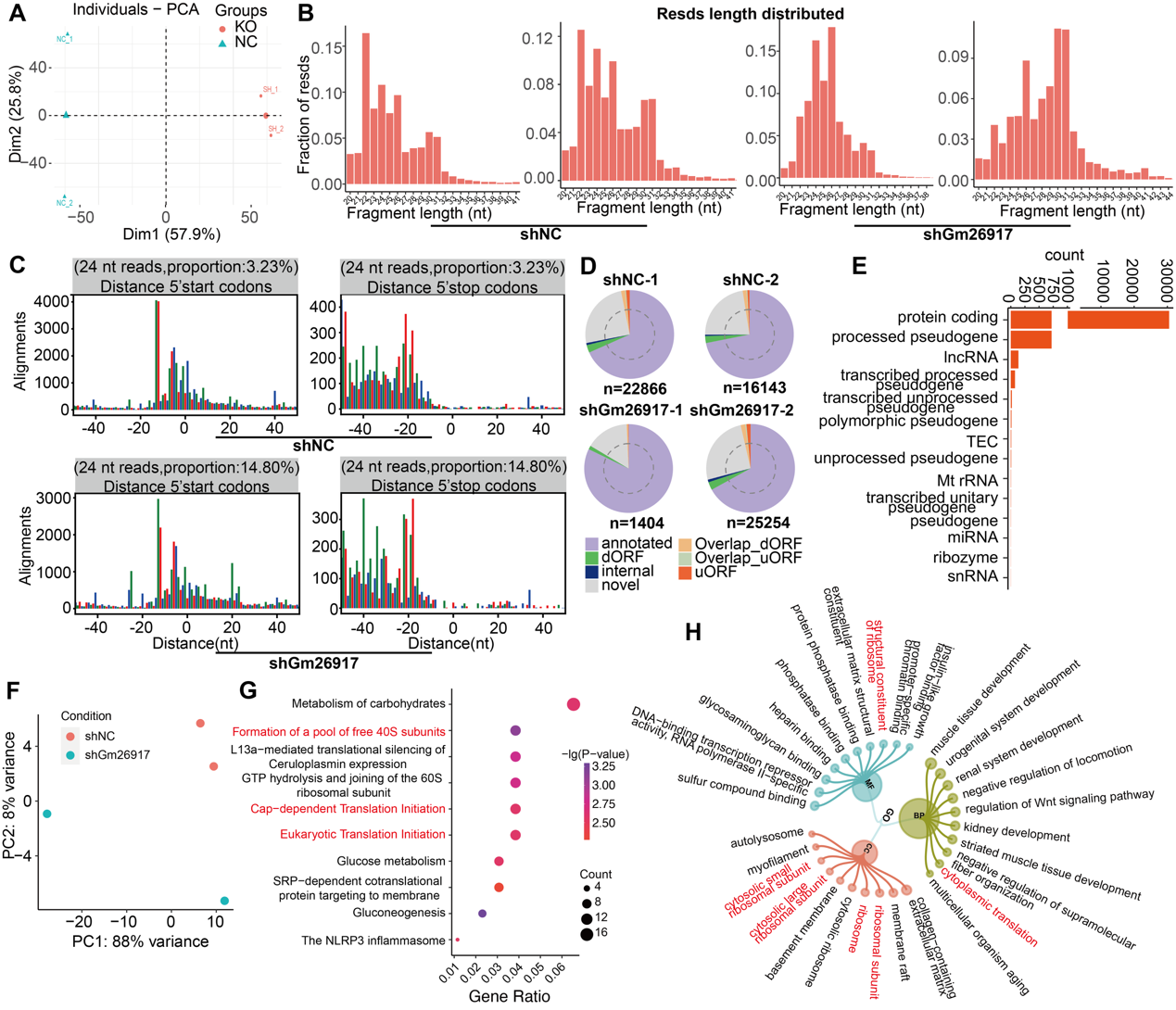


**Figure S7. RNA-seq and Ribo-seq quality control. A)** PCA of shNC and shGm26917 Ribo-seq data. **B)** Read length distribution profiles. **C)** Ribosome footprint (24nt) density around start/stop codons. **D)** ORF number and type classification. **E)** Gene category distribution of ORFs. **F)** PCA of shNC and shGm26917 RNA-seq data. **G and H)** Pathway enrichment analysis of TE-altered genes: Reactome (G) and GO. H) Data: mean ± SD (n=2). Statistical thresholds as in Figure 7.


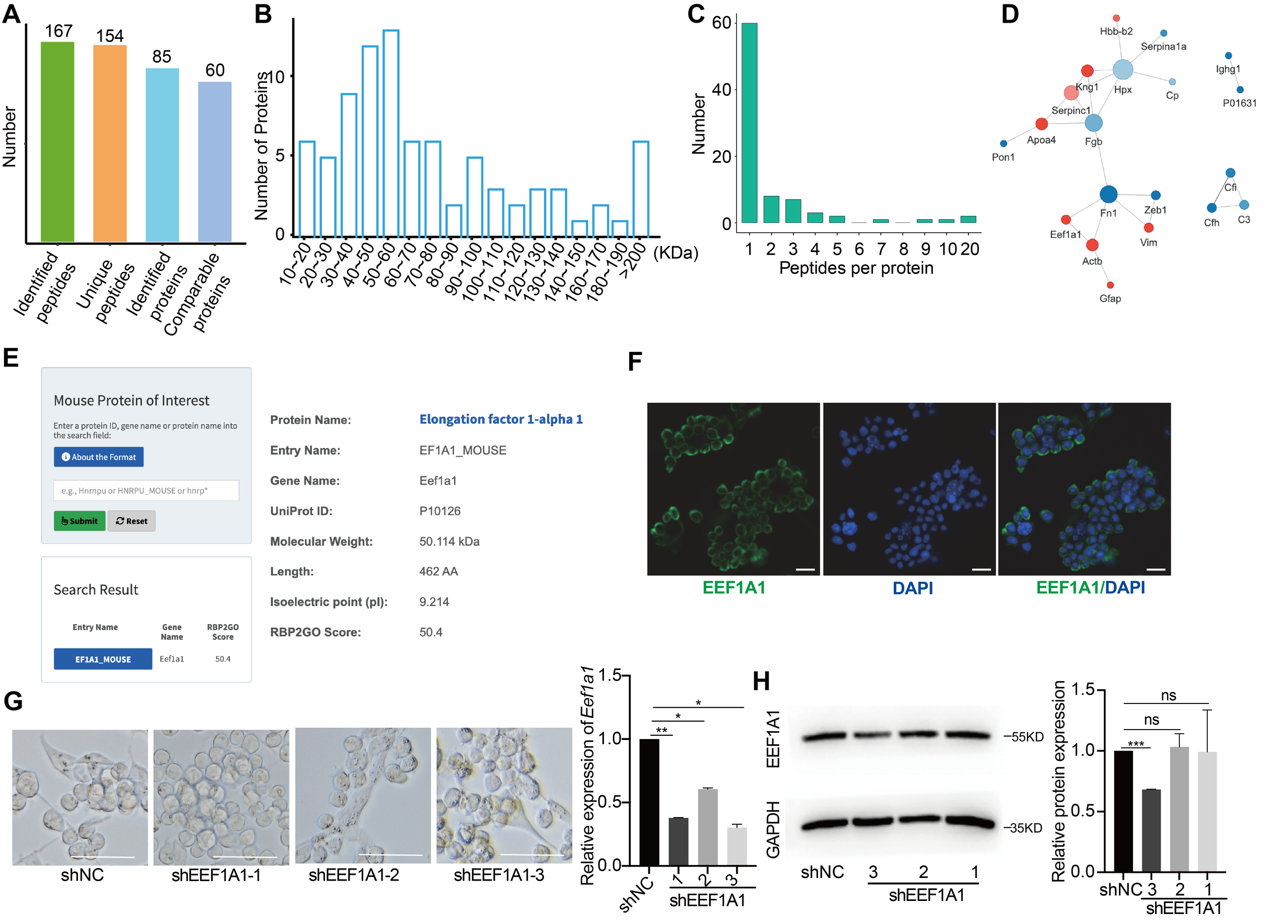


**Figure S8. RAP-MS characterization of EEF1A1 interactome. A)** Protein identification statistics from RAP-MS. **B)** Molecular weight distribution of detected proteins. **C)** Peptide counts per protein. **D)** Protein-protein interaction network analysis. **E)** **The prediction RBP score of EEF1A1.** **F)** Immunofluorescence localization of EEF1A1 in FGSCs. **G)** FGSC morphology (three independent shRNAs) and *Eef1a1* knockdown efficiency detected by qRT-PCR. **H)** Western blotting confirmation of *Eef1a1* knockdown. Scale bars: 50 μm. *p<0.05, **p<0.01; ns: not significant (mean ± SD; n=3).

**Table S1. Sequences of the primer pairs used for PCR analysis**

| **RT-PCR** |  |
| --- | --- |
| *Oct4* F | CACCATCTGTCGCTTCGAGG |
| *Oct4* R | AGGGTCTCCGATTTGCATATCT |
| *Ddx4* F | GGAAACCAGCAGCAAGTGAT |
| *Ddx4* R | TGGAGTCCTCATCCTCTGG |
| *Fragilis* F | GTTATCACCATTGTTAGTGTCATC |
| Fragilis R | AATGAGTGTTACACCTGCGTG |
| *Blimp1* F | CGGAAAGCAACCCAAAGCAATAC |
| *Blimp1* F | CCTCGGAACCATAGGAAACATTC |
| *Sycp3* F | AGCCAGTAACCAGAAAATTGAGC |
| Sycp3 R | CCACTGCTGCAACACATTCATA |
| *Stra8* F | ACCCTGGTAGGGCTCTTCAA |
| *Stra8* F | GACCTCCTCTAAGCTGTTGGG |
| DDX4HA F1 | GGCAAGCACACGTTGAATAC |
| DDX4HA F2 | CATGAAGTGCAAGAACGTGG |
| DDX4HA R | GGTTGGGAGGAAGAACAGAAG |
| *Nat10* Loxp F | GGAACCATGAGTATTGTAGCCTGC |
| *Nat10* Loxp R | CTATTGGCTGTGACTTCAGCAGAC |
| *Gm26917* sanger F | AGTTCACGGTGGGTTCGTC |
| *Gm26917* sanger R | GAGACAGTCAAACCACGCG |
| *Gapdh* F | TGGATTTGGACGCATTGGTC |
| *Gapdh* R | TTTGCACTGGTACGTGTTGAT |
|  |  |
| **RT-qPCR** |  |
| *Sycp3* F | GTTGCTGATGAAAAGGCTCCA |
| Sycp3 R | TTCGAACATTTGCCATCTCTTGC |
| *Stra8* F | GTTTGCCACCTGCAACTCAG |
| *Stra8* F | TCCAGGCACTTCAGCAACAT |
| *Nat10* F | ATGAATCGGAAGAAGGTG |
| *Nat10* R | CTTTTCCTCGATCCCCA |
| *Gm26917* F | GCAATTATTCCCCATGAACG |
| *Gm26917* R | GGCCTCACTAAACCATCCAA |
| *Gm42418* F | TCGCACGCCCCCGTGGC |
| *Gm42418* R | TCACTACCTCCCCGGGTC |
| *Malatl* F | TTTTGAGGGCTGACTGCCAA |
| *Malatl* R | GGTTGTGCTGGCTCTACCAT |
| *Fndc3a* F | TCCGTAGTAATCATGGCGGC |
| *Fndc3a* R | TTGTTGGGGAGGGGTGTTTC |
| *Sox6* F | GCTGCTTTTGTTTGGCAGGT |
| Sox6 R | GCCTGTGAAGTCCCCAACAT |
| *Dock2* F | ACTGCTGCTCTTTCTTACCCA |
| *Dock2* R | GGCATTGAGATGAGCCAACA |
| *Rpl10* F | TTGGGACGGAAGAAAGCAAA |
| Rpl10 R | GTTGGCACAAATACGGGCAG |
| *Cmip* F | TGCAGAAGATCCAAGACAGGAA |
| *Cmip* R | CAAACAGCTCCCCGTCATCA |
| *Hyou1* F | GGGCGCCAATGAGCGT |
| *Hyou1* R | TTCCTCCGAGATTCCTTGTTCA |
| *Bcl2* F | CTTTCTCCTCCTCCGCTGC |
| *Bcl2* R | CAACACCGAGGTGCCCAG |
| *Etv4* F | CATTCCCAGATGATGTCTGCAT |
| *Etv4* R | CCACAGTTGTAAGGCACCCC |
| *Esr1* F | TTGAACCAGCAGGGTGGC |
| *Esr1* R | AGGCTTTGGTGTGAAGGGTC |
| *Akt3* F | AGGAAAGAGAAGAGTGGACGG |
| *Akt3* R | TGGGTTGTAGACGCATCCAT |
| *Gapdh* F | AGGTCGGTGTGAACGGATTTG |
| *Gapdh* R | TGTAGACCATGTAGTTGAGGTCA |
|  |  |
| **acRIP-qPCR** |  |
| A *etv4* F | CCTTTCATCCTCCGCTCCAT |
| A *etv4* R | GCTGTCCGATCCAAAGCC |
| A *esr1* F | CTCAACAGCGTGTCGCCTA |
| A *esr1* R | TCTCCAGGTAGTAGGGCACC |
| A *akt3* F | TTGCCTGTAATGCACTACCA |
| A *akt3* R | TTCTCGGAGCTGACTTCTTG |
| A *Gm26917* F | TTGCGCCTCGCTGCTGT |
| A *Gm26917* R | CGACGCGACACAACGCCA |
| A *Gm42418* F | GGCTTTTCTACGTTGGCTGG |
| A *Gm42418* R | AGCTAGGTACCCGGGACAG |
| A *malat*1 F | AGGCAGGCAAATCTCTGTGA |
| A *malat1* R | CTTGTAGCCCCTGCTGTCTT |
|  |  |
| **Ribo qPCR** |  |
| *RPL3* ATG A | agcggataacaatttcacacaggcaggaaacagctatgacGAGAATTTCCTGTGAG |
| *RPL3* ATG BF | GCGTGTGGCGGCGAGATGTC |
| *RPS17* ATG A | agcggataacaatttcacacaggcaggaaacagctatgacTTGGTGCGAACGCGGC |
| *RPS17* ATG BF | CCGAGACCCGCCAACATGGG |
| *klf4* ATG A | agcggataacaatttcacacaggcaggaaacagctatgacTCGCCAGGTGGCTGCC |
| *klf4* ATG BF | TGGGCCCCCACATTAATGAG |
| *pgam1* ATG A | agcggataacaatttcacacaggcaggaaacagctatgacACCAGCTTGTAGGCAG |
| *pgam1* ATG BF | CCTCTGCCCGTCGCCATGGC |
| *E2F7* ATG A | agcggataacaatttcacacaggcaggaaacagctatgacGTCCAAGGTCTGCTCG |
| *E2F7*  ATG BF | TGAAGGAGCTGATGAATGCC |
| *FGF10* ATG A | agcggataacaatttcacacaggcaggaaacagctatgacTGTCTCATCAGAAGGA |
| *FGF10* ATG BF | TCTTACCCTTCCAGTATGTT |
| *Gapdh*-ATG A | agcggataacaatttcacacaggcaggaaacagctatgacCCGGGGTAAGGGCAGC |
| *Gapdh*-ATG BF | AACCCTTAAGAGGGATGCT |
| universal BR | agcggataacaatttcacacagg |
|  |  |
| **CLIP-qPCR** |  |
| *Rpl10* F1 | TCTCTTTTCCTCTGGCGC |
| *Rpl10* F2 | TGGTGAGTGCTGAACC |
| *Rpl10* F3 | TGAGGCTGTGGGTGG |
| *Rpl10* F4 | AAGTCTCGTTTCTGCCGT |
| *Rpl10* F5 | AAGAAAGCAAAAGTTGAT |
| *Rpl10* F6 | CGAGACGGTTTCTCT |
| *Rpl10* F7 | ATGGTAAAGAGTTGTGGC |
| *Rpl10* F8 | AAGCCCCAGGGCACA |
| *Rpl10* F9 | TGGTCCTCCTTGCTG |
| *Rpl10* F10 | GCTGTGGTGTCAAATATA |

**Table S2. Information of antibodies**

| **REAGENT or RESOURCES** | **SOURCE** | **IDENTIFIER** |
| --- | --- | --- |
| **Antibodies** |  |  |
| Rabbit monoclonal to Stra8 | Abcam | Cat# ab308125 |
| Rabbit polyclonal anti-DDX4 | Abcam | Cat# ab13840 |
| Rabbit monoclonal to Cyclin A2 | Abcam | Cat# ab181591 |
| Rabbit monoclonal to Cyclin B1 | Abcam | Cat# ab181593 |
| Rabbit monoclonal to Cyclin D1 | Abcam | Cat# ab134175 |
| Rabbit monoclonal to N4-acetylcytidine (ac4C) | Abcam | Cat# ab252215 |
| EEF1A1 Antibody | abmart | Cat# T58782 |
| Puromycin Rabbit pAb | ABclonal | Cat# A21205 |
| PARP Antibody | Cell Signaling Technology | Cat# 9542t |
| Cleaved Caspase-3 (Asp175) (5A1E) Rabbit mAb | Cell Signaling Technology | Cat# 9664s |
| BAX Antibody | Cell Signaling Technology | Cat# 2772t |
| Bcl-2 (D17C4) Rabbit mAb | Cell Signaling Technology | Cat# 3498t |
| RPL10 Antibody | Cell Signaling Technology | Cat# 72912s |
| Rabbit (DA1E) mAb IgG XP® Isotype Control | Cell Signaling Technology | Cat# 3900s |
| Mouse monoclonal anti-OCT4 | Santa Cruz Biotechnology | Cat# sc-9081 |
| SYCP3 | Santa Cruz Biotechnology | Cat# sc-20845 |
| Mouse monoclonal anti-GAPDH | Proteintech | Cat# 60004-1-Ig |
| NAT10 Polyclonal antibody | proteintech | Cat# 13365-1-ap |
| CDK4 Polyclonal antibody | proteintech | Cat# 11026-1-ap |
| Beta Tubulin Polyclonal antibody | proteintech | Cat# 10094-1-AP |
| [HRP-conjugated Goat Anti-Rabbit IgG(H+L)](https://www.ptgcn.com/products/HRP-conjugated-Affinipure-Goat-Anti-Rabbit-IgG-H-L-secondary-antibody.htm) | Proteintech | Cat# SA00001-2 |
| HRP-conjugated Goat Anti-Mouse IgG(H+L) | proteintech | Cat# SA00001-1 |
| CoraLite488-conjugated Goat Anti-Rabbit IgG(H+L) | proteintech | Cat# SA00013-2 |
| CoraLite594 – conjugated Goat Anti-Rabbit IgG(H+L) | Proteintech | Cat# SA00013-4 |
| CoraLite488-conjugated Goat Anti-Mouse IgG(H+L) | proteintech | Cat# SA00013-1 |
| CoraLite594 – conjugated Goat Anti-Mouse IgG(H+L) | proteintech | Cat# SA00013-3 |

**Table S3. Sequences of shRNA and a negative control shRNA**

| **shNAT10** |  |  |
| --- | --- | --- |
| Y24155 | *Nat10* | TTGCTGTTCACCCAGATTATC |
| Y24156 | *Nat10* | AGAGTGGGACCTTGAACTTAA |
| Y24157 | *Nat10* | GCAGTGGAGAAGTGGCTTAAT |
| GL427NC2 | NC2 | CCTAAGGTTAAGTCGCCCTCG |
| **shGm26917** |  |  |
| Y33073 | *Gm26917* | GCGTTTGCTCTCTCGTCTA |
| Y33074 | *Gm26917* | GCTCCAATAGCGTATATTA |
| Y33075 | *Gm26917* | GCGATTTGTCTGGTTAATT |
| GL401NC | NC | TTCTCCGAACGTGTCACGT |
|  |  |  |
|  |  |  |
| **shRPL10** |  |  |
| shRNA618 | *Rpl10* | GATGGCTGTGGTGTCAAATAT |
| shRNA193 | *Rpl10* | ATATGGTGTCAGATGAATATG |
| shRNA281 | *Rpl10* | TGGCAAGGATGGCTTTCATAT |
| NC |  | TTCTCCGAACGTGTCACGT |
| **shEEF1A1** |  |  |
| Y41120 | *Eefla1* | GCGTGGTATCACTATTGACAT |
| Y41121 | *Eefla1* | CGTTCTGGTAAGAAGCTGGAA |
| Y41122 | *Eefla1* | CCAGTCAATGTAACAACTGAA |
| GL401NC | NC | TTCTCCGAACGTGTCACGT |

**Table S4. Probe sequences of RAP**

| gene name | speces | labelling pattern | Probe sequence | sequence site | nmol |
| --- | --- | --- | --- | --- | --- |
| Gm26917 | mouse | 5-terminal biotin | CTTCTCAGACACAAACGGGAAGGCACACAG | 475-504 | 3.36 |
| Gm26917 | mouse | 5-terminal biotin | AAGGTTCTCTTCCAAGGGCATTCTGAGCAT | 734-763 | 3.52 |
| Gm26917 | mouse | 5-terminal biotin | GGAAGATAGCTAGAGAAGGAAACTTTCTCACTGAGGGCGG | 881-920 | 2.46 |
| Gm26917 | mouse | 5-terminal biotin | GATCAATCAACGCCAGACACGGACCCTCTC | 1471-1500 | 3.52 |
| Gm26917 | mouse | 5-terminal biotin | GTAGCACCATTCACTCCCCACACCAACGAC | 2900-2929 | 3.59 |
| Gm26917 | mouse | 5-terminal biotin | CTCTCCGACACCTCTCTTATCCGCTCTCCC | 3090-3119 | 4.04 |
| Gm26917 | mouse | 5-terminal biotin | CACAGTTATCCAAGTAGGAGAGGAGCGAGC | 3482-3511 | 3.24 |
| Gm26917 | mouse | 5-terminal biotin | ATCGGCCCGAGGTTATCTAGAGTCACCAAA | 3649-3678 | 3.36 |
| Gm26917 | mouse | 5-terminal biotin | CCCCTTCTCTCACCTCACTCCAGACACCTC | 5311-5340 | 3.96 |
| Lac Z | E.coli | 5-terminal biotin | GGGTTTTCCCAGTCACGACGTTGTAAAACGACGGCCA | 1-37 | 1.47 |
| Lac Z | E.coli | 5-terminal biotin | CGAAAGGGGGATGTGCTGCAAGGCGATTAAGTTGGGT | 45-81 | 1.51 |
| Lac Z | E.coli | 5-terminal biotin | GGAAGGGCGATCGGTGCGGGCCTCTTCGCTATTACGC | 89-125 | 2.92 |
| Lac Z | E.coli | 5-terminal biotin | TACCGCATCAGGCGCCATTCGCCATTCAGGCTGCGCA | 133-169 | 1.57 |
| Lac Z | E.coli | 5-terminal biotin | ACCATATGCGGTGTGAAATACCGCACAGATGCGTAAG | 177-213 | 1.52 |

**Table S5. Probe sequences of FISH**

| Gm26917_mus |  |  |  |
| --- | --- | --- | --- |
| sequence number | probe sequence | initiation site | GC% |
| 1 | accactgagaaaagtgcgcg | 192 | 55.00% |
| 2 | cacacatccacaaggaccac | 216 | 55.00% |
| 3 | agtgaaacacgtgagggcac | 248 | 55.00% |
| 4 | gacatggtcaagcgagacac | 274 | 55.00% |
| 5 | agacgggaagggtatgcaac | 322 | 55.00% |
| 6 | cacctcgacgcttacaagaa | 360 | 50.00% |
| 7 | cacaccagggaatgggaagc | 436 | 60.00% |
| 8 | cacaaacgggaaggcacaca | 476 | 55.00% |
| 9 | gagaaactttccaaggccag | 630 | 50.00% |
| 10 | ccacgggaaagcaatgagtc | 654 | 55.00% |
| 11 | gaaagccaggcctctcaaag | 678 | 55.00% |
| 12 | caggaaggttctcttccaag | 748 | 50.00% |
| 13 | acagggaaaccagaagacca | 802 | 50.00% |
| 14 | aagagaggatgcatgcgagg | 827 | 55.00% |
| 15 | aaactttctcactgagggcg | 882 | 50.00% |
| 16 | ctttccggaagatagctaga | 907 | 45.00% |
| 17 | tcaagaccgtaagaagcccg | 931 | 55.00% |
| 18 | cgtggtagacgagagagcaa | 1015 | 55.00% |
| 19 | aacggcaggacgacagacag | 1085 | 60.00% |
| 20 | cacacaaccggagccacatg | 1115 | 60.00% |
| 21 | aaagtcgtggacgcgagcga | 1301 | 60.00% |
| 22 | gaggcgacacaaccacacag | 1379 | 60.00% |
| 23 | cgagatcaatcaacgccaga | 1484 | 50.00% |
| 24 | cgatgcctaccgacgaagac | 1592 | 60.00% |
| 25 | gaggcgacccgaaacacgac | 1672 | 65.00% |
| 26 | aaaaccggcgggaatcacac | 1733 | 55.00% |
| 27 | ctcacaagggaccgagagac | 1788 | 60.00% |
| 28 | gggaacgacacagcagaacg | 1863 | 60.00% |
| 29 | gggacaatgaccactgctag | 2174 | 55.00% |
| 30 | agagggaggaacgaggggag | 2415 | 65.00% |
| 31 | tcgacgagcgacttgaaccc | 2439 | 60.00% |
| 32 | aggacgaacccaccgtgaac | 2506 | 60.00% |
| 33 | cgaagccggcgagagaagac | 2592 | 65.00% |
| 34 | aacgtcggggaaccgggaag | 2645 | 65.00% |
| 35 | aactgaccgcggcgctaaac | 2749 | 60.00% |
| 36 | aatgaccggtagcaccattc | 2918 | 50.00% |
| 37 | cgagacagtcaaaccacgcg | 2944 | 60.00% |
| 38 | cggaaagagaagcgcgacac | 2967 | 60.00% |
| 39 | ctgtcggggtccgacaaaac | 3175 | 60.00% |
| 40 | cggaacgggagagtgcatgc | 3208 | 65.00% |
| 41 | gaggacaaaccgggggtgag | 3245 | 65.00% |
| 42 | cacacacaagacggggagag | 5240 | 60.00% |
| 43 | gacgggaaagaggcacgacg | 5455 | 65.00% |
| 44 | cctcgtagacacggaagagc | 5477 | 60.00% |
| 45 | cgggtcaaaaacccgtaacg | 5507 | 55.00% |
| 46 | gccaacgtagaaaagccaga | 5591 | 50.00% |
| 47 | gacacgcgaggccagaaaag | 5677 | 60.00% |
| 48 | ggaacgcgacagctaggtac | 5716 | 60.00% |
|  |  |  |  |
| Rpl10_mus |  |  |  |
| sequence number | probe sequence | initiation site | GC% |
| 1 | gacaataggatcgtcggtgg | 19 | 55.00% |
| 2 | ggagaactattgaagccttc | 685 | 45.00% |
| 3 | attgaacgatttggtagggt | 708 | 40.00% |
| 4 | gcgaacttgatgcgagattt | 732 | 45.00% |
