# Supplementary material for: N4‐acetylcytidine in LncRNA Gm26917 Promotes Translation in Female Germline Stem Cells by Recruiting Ribosomal Protein mRNA via EEF1A1
Source: Adv Sci (Weinh). 2026 Mar 17;13(30):e20059. doi: 10.1002/advs.202520059 (PMC13248794; doi:10.1002/advs.202520059)
Supplement: Supplementary file 2 — Supporting File: advs74863‐sup‐0002‐blots.docx. [file ADVS-13-e20059-s001.docx]

**Fig 1D Fig 1E Fig 1F**


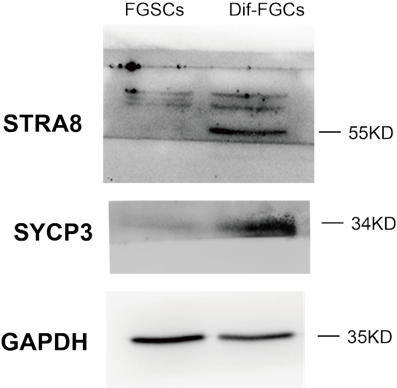

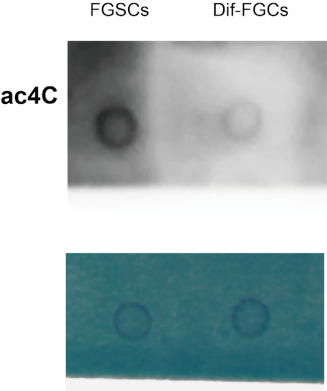
**
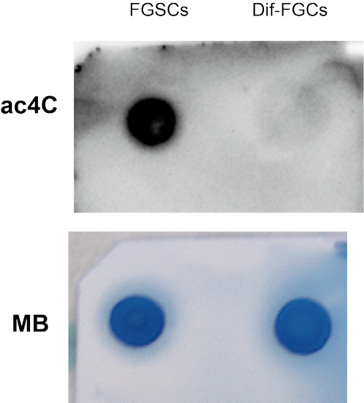
**


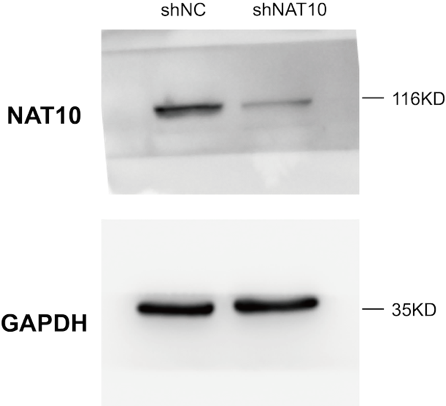

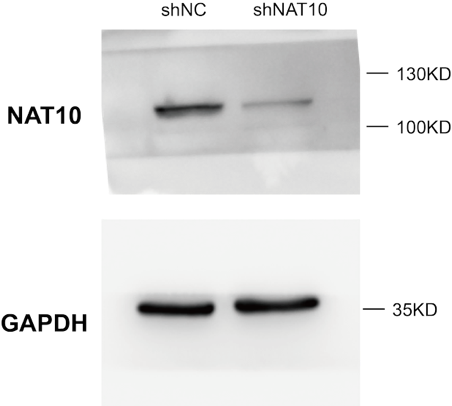

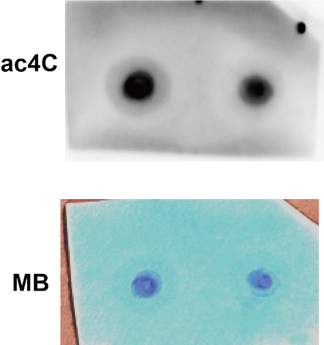
**Fig 1H Fig 2B Fig 2C**


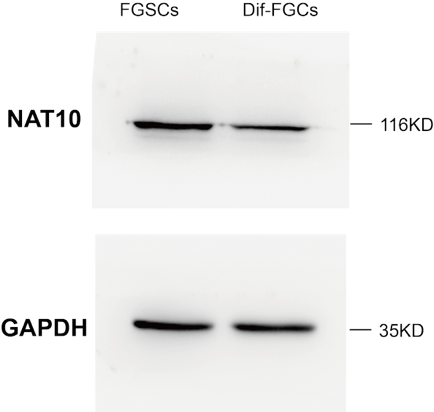


**Fig 2G Fig 2L Fig 2M**

**
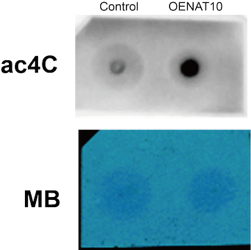

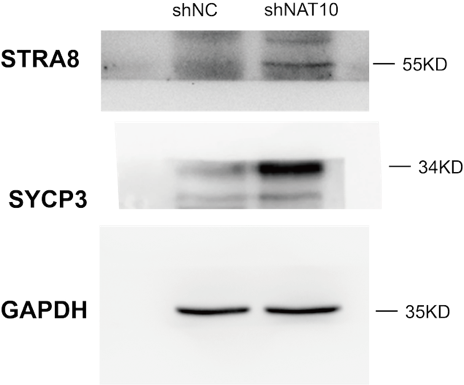

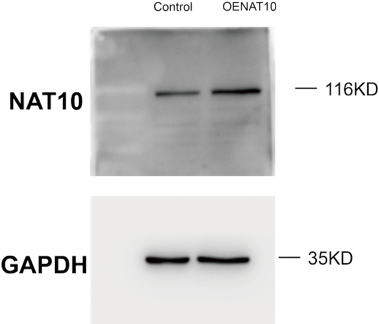
**

**
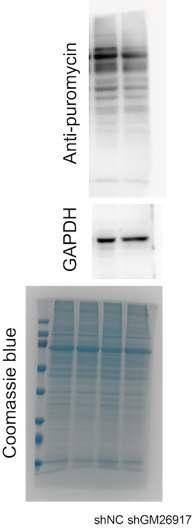
**
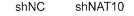
**
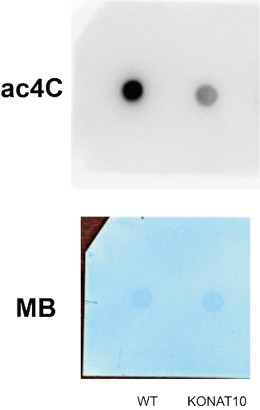
Fig 3F Fig 4P Fig 6D**

**
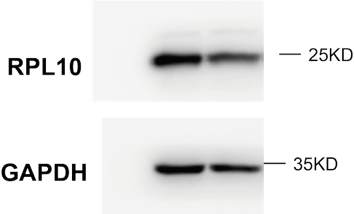

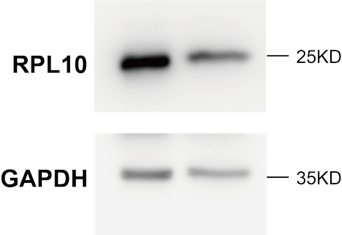
**

**Fig 6H**

**
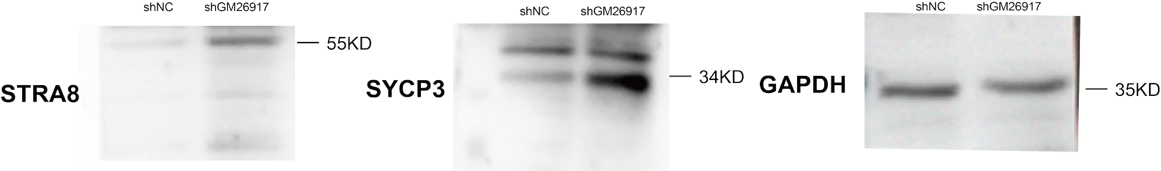
**

**Fig 6J Fig 6M**

**
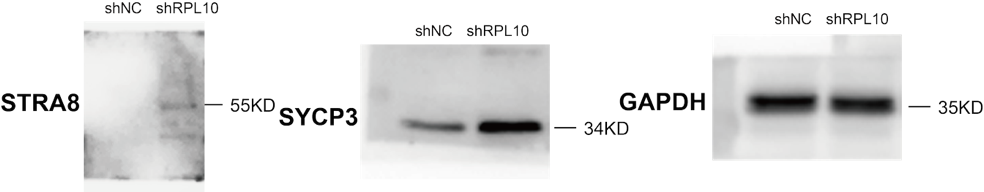

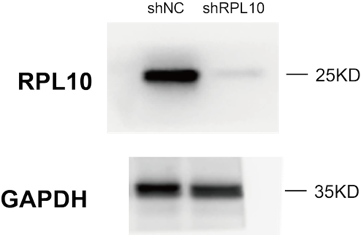
**

**
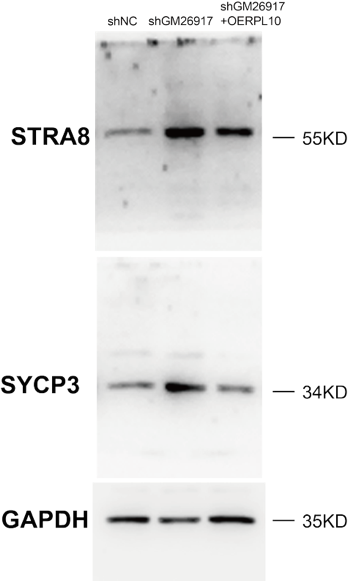
Fig 6O Fig 6R**

**
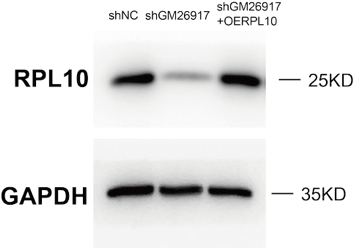
**

**Fig 7A Fig 7B Fig 7C**


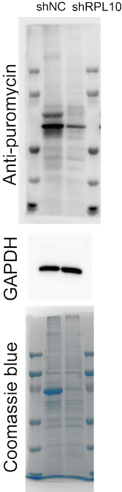
**
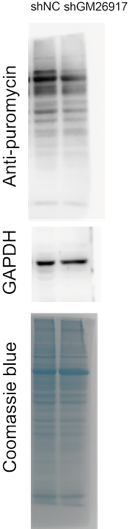
** **
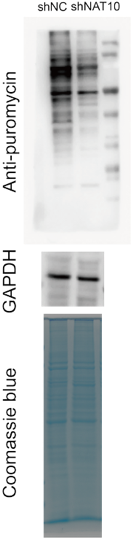
**

**
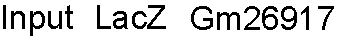
Fig 8C Fig 8D**

**
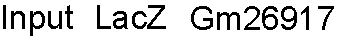

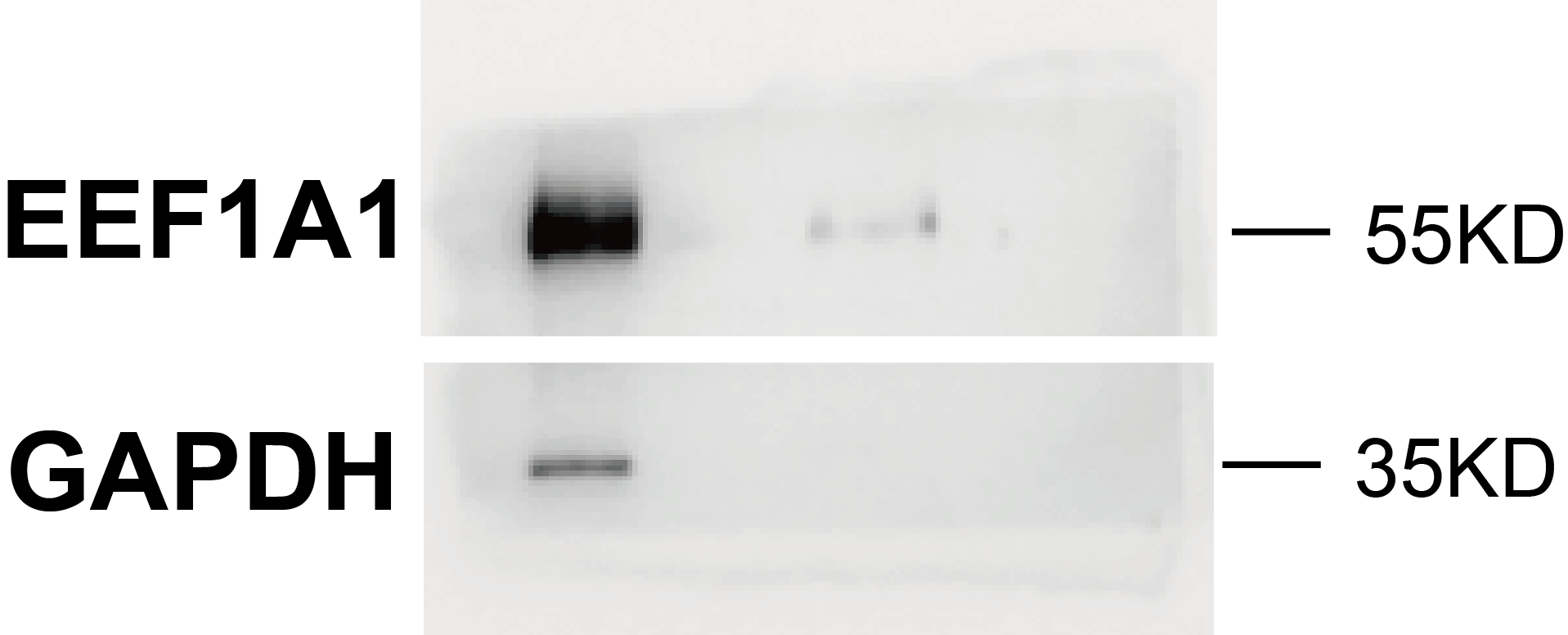

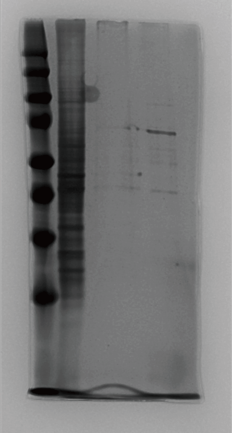
**

**Fig 8N Fig 8S**

**
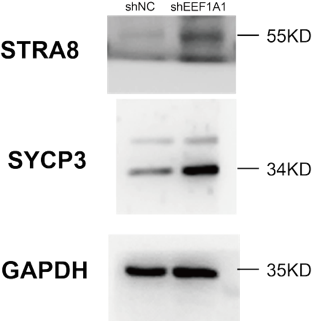

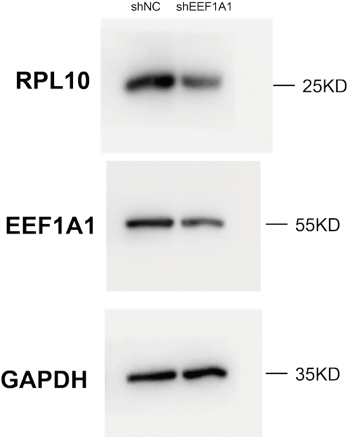
**

**
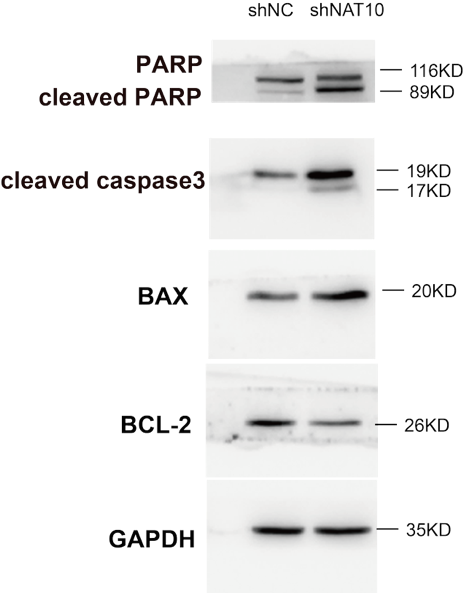

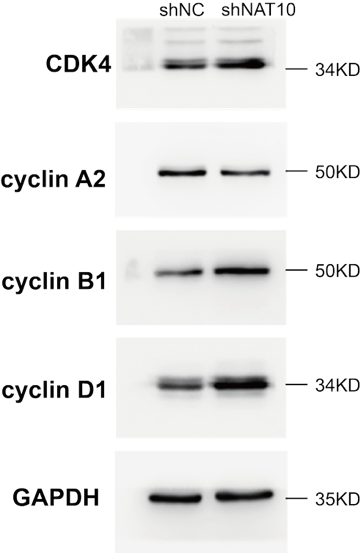
Fig S2D Fig S3C Fig S3D**

**
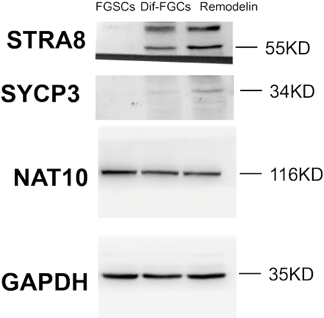
**

**Fig S6C Fig S6E**


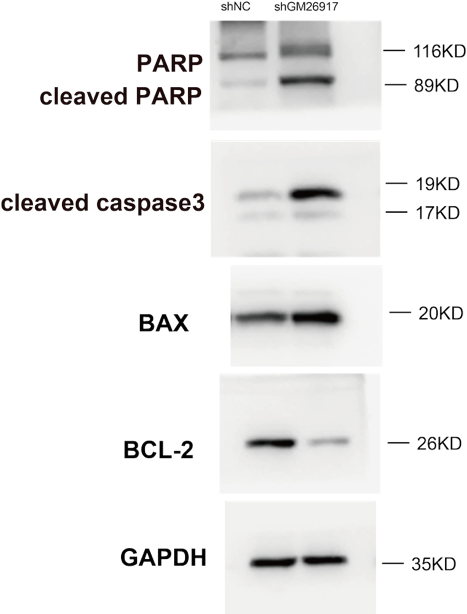

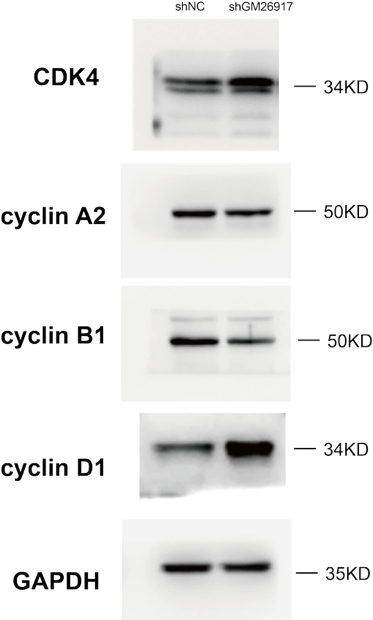


**Fig S6H Fig S6J**


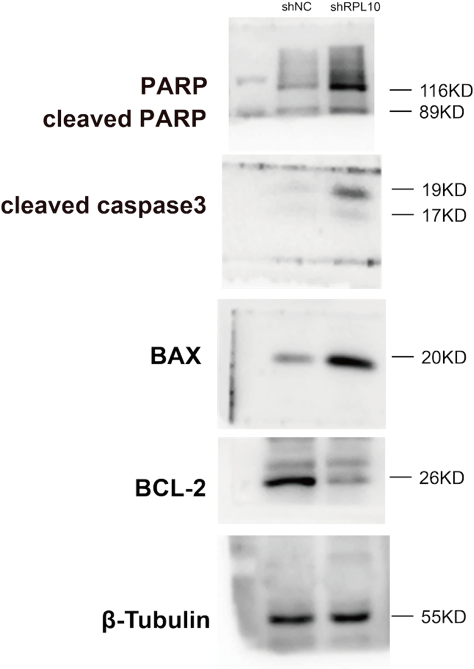

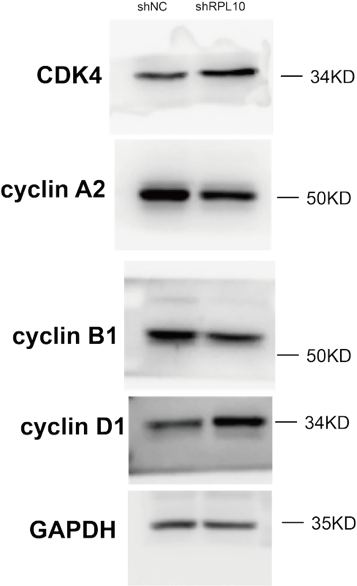


**Fig S8H**

**
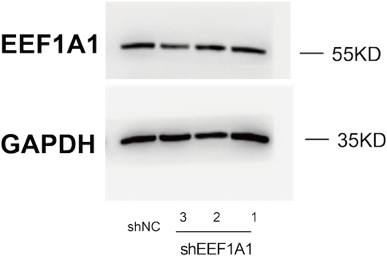
**
